# Targeting the tumor microenvironment: reprogramming macrophages as a novel therapeutic strategy in FUOM-deficient glioblastoma

**DOI:** 10.1038/s41419-026-08701-5

**Published:** 2026-04-09

**Authors:** Bing Lu, Manyu Xu, Hui Zhang, Xiaojin Zhang, Pingping Sun, Lei Yang, Jinlong Shi, Jianfei Huang

**Affiliations:** 1 Clinical and Translational Research Center & Institute of Oncology, Affiliated Hospital of Nantong University, Nantong, P.R. China; 2 Department of Pathology, Jinling Hospital, Affiliated Hospital of Medical School, Nanjing University, Nanjing, P.R. China; 3 Medical School of Nantong University, Nantong, P.R. China; 4 Department of Neurosurgery, Affiliated Hospital of Nantong University, Nantong, P.R. China

**Keywords:** Biomarkers, Cancer

## Abstract

Glioma, the most prevalent CNS malignancy and generally poor prognosis, is characterized by a highly immunosuppressive tumor microenvironment. Fucose mutarotase (FUOM) is a known rockulose mutase secretion protein involved in pathological immune remodeling. However, the precise role of FUOM involvement in glioma has yet to be elucidated. Herein, we delineated FUOM expression using multiplex-immunohistochemistry on tissue microarrays, and its prognostic predictive value was assessed using the Cox regression method. Then we investigated the altered proliferation, migration, and invasion capabilities of glioma cells upon regulated FUOM expression in vitro. Chemokine antibody microarray, immunoassay, and Co-IP were employed to detect interactions between macrophages and glioma cells. In addition, the in vivo therapeutic effects of FUOM were confirmed using animal subcutaneous glioma models. FUOM was highly expressed in glioma tissues and correlated with aggressive glioma progression and unfavorable patient prognosis. Macrophage infiltration into the glioma TME was observed upon FUOM downregulation, with induced CXC motif chemokine ligand-13 (CXCL13) release in maintaining M2-like phenotype. In addition, conditioned media from FUOM knockdown glioma cell lines induced M2-like macrophage chemotaxis migration. Finally, blocking FUOM expression on glioma models enhanced M2-like macrophage phenotype and increased chemotactic migration both in vivo and in vitro. Collectively, our work reveals that FUOM induces M2-like macrophage polarization and promotes glioma progression by mediating CXCL13 secretion.

## Introduction

Glioma is the most common type of primary intracranial tumor, representing over 80% of malignant primary brain in the central nervous system (CNS) [1]. The World Health Organization (WHO) classifies gliomas into four grades, with different overall survival times. Grade 1–2 are low-grade gliomas (LGGs) with median survival typically ranging from 8 to 10 years. For Grade 3 gliomas: IDH-wildtype has an average survival of 2–3 years; IDH-mutant averages 3–5 years; and IDH-mutant with 1p/19q codeletion (anaplastic oligodendroglioma) averages 4–9 years. Grade 4 gliomas (e.g., glioblastoma, GBM) have the shortest median survival of approximately 15–16 months. Approximately half of all diffuse gliomas are classified as glioblastomas (Grade 4) based on the 2021 WHO classification system. The remaining low-grade or lower-grade gliomas (LGGs) are classified by histology and molecular characteristics.

Glioma cells exhibit robust resistance to established therapeutic approaches, and the immunosuppressive nature of their tumor microenvironment (TME) significantly compromises the pathogen elimination capabilities of the central nervous system (CNS) [2]. Molecular markers are important in determining the trajectory of glioma cell behavior and have been explored as potential therapeutic targets in clinical trials. Nonetheless, the success rate of these interventions remains limited. Therefore, a deeper understanding of the mechanisms driving glioma progression and the identification of new molecular targets for glioma treatment are urgently required [3].

Despite the potential of immunotherapy in glioma treatment, its efficacy remains unsatisfactory, urging the need for in-depth studies into the genetics and biological functions of immune cells that infiltrate glioma [4]. The brain’s unique immunological profile, particularly the role of myeloid cell populations in the immune microenvironment, contributes significantly to the establishment of a suppressive milieu [2, 5]. A key feature of the glioma TME is the predominance of infiltrating immune cells, which markedly influence tumor progression. Notably, the glioma TME is characterized by a lack of lymphocytes and a predominance of immunosuppressive myeloid cells [6]. Recent evidence highlights the ability of tissue-resident macrophages to penetrate, proliferate within, and effectively function in the CNS, which can express T-cell co-stimulatory cytokines when activated [7]. Among the various immune cells in the TME [8], tumor-associated macrophages (TAMs) are the most abundant and play a pivotal role in glioma development and progression [9, 10]. TAMs are broadly categorized into two types: M1 and M2-like macrophages [11]. M1-like macrophages are characterized by their production of pro-inflammatory cytokines, their ability to present antigens to immune cells, and their capacity to engulf tumor cells [12]. In contrast, M2-like macrophages secrete immunosuppressive cytokines, thereby facilitating tumor progression. On the other hand, M2-like macrophages play crucial roles in tissue repair, immune resolution, and modulation of excessive inflammation, thereby contributing to the maintenance of a more balanced tumor microenvironment [12].

The function and composition of TME can be shaped by both intrinsic secretory factors of cancer cells and immune cell signaling [13]. Fucose mutarotase (FUOM) is a rockulose mutase secretion protein involved in protein glycosylation [14]. Its presence in the cytoplasm or secretion into biological fluids is believed to be pivotal in cell-cell adhesion and recognition. However, the specific mechanisms through which FUOM influences glioma progression remain unclear. Additionally, considering that cytokines play a significant role in mediating tumor progression and immune evasion of cancer cells [15], targeting these immunosuppressive cells and associated signaling pathways presents a promising approach to enhance the effectiveness of immunotherapies and counteract the immune-resistant nature of the TME in glioma [16]. Thus, investigating changes concerning immune cell infiltration and associated cytokine release is essential for advancing immunotherapeutic strategies and identifying potential biomarkers for optimal therapeutic classification [17]. Moreover, the interactions between macrophages and tumor cells lead to the polarization of M2-like macrophages, induction of chemokine (C-X-C motif) ligand (CXCL), and a dysregulated CXCL chemokine pathway, implicating its role in promoting cancer progression [18]. A reduction in CXCL expression may contribute to the immune evasion of glioma cells and is potentially linked to the efficacy of immune checkpoint inhibitors in patients [19, 20]. However, CXCL13 overexpression on infiltrated M2-like macrophages could imply the aggressive characteristics of macrophages and could provide predictive outcomes for patients diagnosed with malignant astrocytoma [21].

In summary, this study elucidated the prognostic significance of FUOM in glioma patient survival, demonstrating that downregulated FUOM expression facilitates immune evasion of glioma cells through CXCL13-mediated chemotactic interactions, and highlighted the therapeutic potential of targeting the FUOM-CXCL13 axis in triggering M2-like macrophage signaling immune response.

## Materials and methods

### Glioma public patient cohorts

Data on glioma patients were acquired from The Cancer Genome Atlas (TCGA, https://tcga-data.nci.nih.gov/tcga/, last date of access: 2025-10-11, RRID: SCR_003193) and the China Glioma Genome Atlas (CGGA, http://www.cgga.org.cn, last date of access: 2025-10-11). Herein, the TCGA data were used as the primary test group and the CGGA data as the validation set. We accessed gene expression profiles and clinical information for 698 glioma patients and 5 normal individuals from the TCGA database. Patients lacking complete clinical data were omitted from further analysis. Similarly, gene expression data for 693 glioma patients and their clinical information were retrieved from the CGGA database. To facilitate a comprehensive analysis, the gene expression data from both sources were batch-corrected and integrated using the Limma and SVA packages of the R software (version 4.1.2, RRID: SCR_001905).

### Glioma patient specimen

A total of 222 glioma tissue samples, including 193 tumor and 29 non-tumor formalin-fixed paraffin-embedded (FFPE) brain tissue specimens, were obtained from the Affiliated Hospital of Nantong University (Jiangsu, China) from March 2012 to November 2017. FFPE primary glioma tissue blocks were used to construct Tissue Microarrays (TMAs), which consisted of blocks of 2 mm diameter, manually created using the Tissue Microarrayer System Quick Ray (UNITMA, Seoul, Korea) [22]. Serial sections (2 µm thick) of FFPE blocks were cut into TMA sections. Clinical information was retrieved from retrospective data, and the glioma pathological type and WHO grade were determined following the 2021 World Health Organization Classification of Tumors of the Central Nervous System [23]. The IDH1^R132H^ mutation was assessed using immunohistochemistry methods and examined by clinical pathologists. This study included patients with a confirmed pathological diagnosis of primary gliomas who had undergone surgical resections with/without chemotherapy and/or radiotherapy, comprising 113 men and 80 women, with a median age of 53.79 ± 13.03 years (ranging from 21 to 86 years) and a median 5-year survival time of 26.94 ± 26.78 months. Among them, 24 patients had chemotherapy after surgical resection, and 38 patients had radiotherapy after surgical resection. We have followed the REMARK reporting guidelines during the whole procedure, and the ethical approval for using human samples in this study was obtained from the Human Research Ethics Committee of the Affiliated Hospital of Nantong University (Grant No. 2018-K020).

### Multiplex-immunohistochemistry (mIHC) and immunohistochemistry (IHC) staining analysis

The mIHC staining was performed using the Opal 7-Color Manual IHC Kit (NEL810001KT, AKOYA Bioscience, MA, USA) as previously described [24]. The process began with deparaffinization and rehydration of the TMA sections. Microwave treatment was then applied using AR6/9 antigen retrieval buffer (AR600250ML/AR900250ML, AKOYA Bioscience, MA, USA). To inhibit endogenous peroxidase activity, the slides were blocked with a tissue-blocking buffer (ARD1A01EA, AKOYA Bioscience, MA, USA). This was followed by incubation with primary antibodies and then with Opal polymer HRP (anti-Ms + Rb) secondary antibody (ARH1001EA, Akoya Bioscience, MA, USA, RRID: AB_2890927). The fluorophores used for visualization were Opal 520, Opal 540, Opal 570, Opal 620, Opal 650, and Opal 690 (AKOYA Bioscience, MA, USA). The multiplex staining involved serial repetitions of the staining cycles, interspersed with microwave treatment steps to detach the antibody complex. The final step involved using Fluor-shield with DAPI (F6057, Sigma, NY, USA) for nuclear visualization. The primary antibodies used included anti-FUOM (1:500, ab188923, Abcam, Cambridge, UK, RRID:AB_2050251), anti-CXCL13 (1:800, MAB801, R&D Systems, MN, USA, RRID:AB_2086049), and anti-GFAP (neighborhoods to tumor cells, 1:10000, ab7260, Abcam, Cambridge, UK, RRID: AB_305808). In the meanwhile, We use panels of immune cell and cytokine markers stained on glioma patient TMAs, anti-CD20 (1:1000, #98708, CST, MIA, USA, RRID: AB_3662710), anti-CD66b (1:2000, ARG66287, Arigobio, ZJ, China, RRID: AB_3662711), anti-CD83 (1:1000, ab244204, Abcam, Cambridge, UK, RRID: AB_3662712), anti-CD3 (1:500, #99940, CST, MIA, USA, RRID: AB_2755035), anti-CD4 (1:500, 67786-1-Ig, Proteintech, WH, China, RRID: AB_2918550),and anti-CD8 (1:500, ab93278, Abcam, Cambridge, UK), anti-CD86 (1:500, #91882, CST, MIA, USA, RRID: AB_2797422), anti-CD25 (1:100, MA5-12680, Invitrogen, CA, USA, RRID: AB_10979943), anti-CD68 (1:500, #76437, CST, MIA, USA, RRID: AB_2799882), anti F4/80 (1:800, ab6640, Abcam, Cambridge, UK, RRID: AB_2131709), anti-CD163 (1:500, #93498, CST, MIA, USA, RRID: AB_2800204), anti-CD206 (1:1000, #24595 T, CST, MIA, USA, RRID: AB_2892682), anti-IL-10 (1:500, ab133575, Abcam, Cambridge, UK, RRID: AB_2894870), and anti-TGF-β (1:500, P1624, Selleck, SH, China, RRID: AB_3717715). Additionally, IHC staining was performed to evaluate IDH1^R132H^ (1:20, DIA-H09, Dianova, HH, GER, RRID: AB_2335716) expression in glioma TMAs, following the manufacturer’s instructions (KIT-9902, MXB Biotechnologies, FJ, China). The immune cell markers used for mIHC staining panels were illustrated in Supplemental Table 1.

Next, the TMAs with DAB and/or fluorescence staining were digitally scanned using the Vectra 3.0 Automated Quantitative Pathology Imaging System (AKOYA Bioscience, MA, USA), and the IHC results were interpreted as H-scores, where intensity values were recorded as 0, 1, 2 and 3, corresponding to unstained, weak, medium and strong positive stains. The total scores were determined by multiplying the staining intensity score by the proportion of positively stained cells, ranging from 0 to 300. The fluorescence intensity of each immune cell marker was quantified utilizing the InForm Tissue Analysis Software (Version 26.1; AKOYA Bioscience, MA, USA). Specific algorithms were designed for the immune cell markers to compute scores automatically based on stained fluorescence intensity using the InForm Software. The protein expression levels of FUOM were assessed as percentage values for each tissue category as follows: the number of positively stained cells divided by the number of stained nuclei, multiplied by the percentage tissue area. The immune scores were calculated as percentage values for each tissue category as follows: the number of positively stained cells divided by the number of stained nuclei, multiplied by 100% of each tissue category (positively stained cell number/nuclei number × 100% of each tissue category).

### Cell lines and culture

Four human glioma cell lines, T98G (RRID: CVCL_0556), U251 (RRID: CVCL_0021), U87mg (RRID: CVCL_0022), and Hs683 (RRID: CVCL_0844), and two macrophage cell lines: THP-1 (human myeloid leukemia mononuclear cell, RRID: CVCL_0006), Raw 264.7 (mouse mononuclear macrophage cell, RRID: CVCL_0493), and C6 rat glioma (RRID: CVCL_0194), together with GL261 mouse glioma (RRID: CVCL_Y003) cell lines were obtained from the National Collection of Authenticated Cell Cultures (CAS, SH, China). The T98G, Hs683, U87mg, and Raw 264.7 cell lines were maintained in vitro using Dulbecco’s Modified Eagle Medium (DMEM) enriched with 10% FBS and 1% penicillin/streptomycin (C0222, Beyotime, SH, China). The U251 cells were cultured in Minimum Essential Medium (MEM) supplemented with 10% FBS, non-essential amino acids (C0332, Beyotime, SH, China), and sodium pyruvate (C0331, Beyotime, SH, China). The THP-1 cells were grown in Roswell Park Memorial Institute (RPMI)-1640 medium, containing 10% FBS and 0.05 mM β-mercaptoethanol (BME, #21985023, Gibco, ThermoFisher, California, USA). C6 rat glioma (RRID: CVCL_0194) and GL261 mouse glioma (RRID: CVCL_Y003) cell lines were cultured in DMEM supplemented with 10% fetal bovine serum (FBS, #04-001-1ACS, Biological Industries, Israel) and antibiotics (100 U/mL penicillin and 100 µg/mL streptomycin). All cell lines were regularly tested for mycoplasma contamination every month and were incubated at 37 °C in a humidified atmosphere with 5% CO_2_.

### Plasmid preparation and cell transfection

Three specific shRNA sequences targeting FUOM (sh-FUOM-a, sh-FUOM-b, sh-FUOM-c) and a negative control (nc) shRNA were acquired from Reinergy (Guangzhou, China). The sequences were: sh-FUOM-a (5′-GATCGTTCTTGCGGACTTGAA-3′), sh-FUOM-b (5′-GGCAAAGATAGAGAGGTTTGA-3′), and sh-FUOM-c (5′-ACGGAAACCTCATCCTCAGGA-3′), with the FUOM-nc plasmid sequence being shRNA-nc (5′-GCTTCGCGCCGTAGTCTTA-3′). A full-length human FUOM plasmid was inserted into a pLVX-IRES-Puro vector for stable overexpression (FUOM-oe), with an empty vector as control (FUOM-oe-nc). Then, the FUOM plasmids were packaged into the Lenti-X™ Bicistronic Expression System (Cat. No. 632183, Puro, Takara Bio), which is designed to constitutively co-express FUOM and/or puromycin resistance when transduced into mammalian cells. Additionally, full-length human CXCL13 was cloned into vector-pcDNA3.1 as CXCL13-oe plasmid, together with an empty vector as control (CXCL13-oe-nc) acquired from Genepharma (SH, China). Glioma cells were transfected with these plasmid constructs using Lipofectamine™ 3000 (L3000001, Invitrogen, CA, USA) following the manufacturer’s guidelines. The culture medium was replaced after 6–8 h of transfection, and the cells were cultured for subsequent experimental analysis.

### Primary glioma model and treatment

#### C6 rat glioma model

Male Wistar rats (RRID: RGD_13508588), aged 4 weeks (weighing 200 ± 21 g), were used for primary glioma implantation were purchased from Nantong Medical University and allowed 1 week of acclimatization to their new surroundings. These rats were housed in temperature-controlled rooms with a 12 h alternating light-dark cycle in the Specific Pathogen Free animal laboratory. Glioma cells were prepared within 10–20 min prior to implantation to prevent a reduction in overall viability. For C6 glioma animal model, the rats were divided into the following 3 groups randomly: (1) negative control group: rats received subcutaneous injection of 20 μL C6 glioma cell suspension, (2) FUOM-sh-c treatment group: rats received subcutaneous injection of 20 μL C6 cells transfected with FUOM-sh-c alone, and (3) FUOM-oe treatment group: rats received subcutaneous injection of 20 μL C6 cells transfected with FUOM-oe plasmids alone (rat *n* = 6, respectively). Briefly, C6 cell suspension (1 × 10^7^) was injected subcutaneously into the left caudate nuclei (1 mm AP, 3 mm ML, −5 mm DV) at the rate of 0.25 μL/min using the stereotactic apparatus (RWD, China). After 2 months of treatment, rats with equivalently sized tumors were sacrificed according to institutional ethical guidelines, and the tumor-bearing brains were collected for histological analysis.

#### GL261 mouse glioma model

For tumor implantation, 3–5 × 10^6^ cells/10 μL GL261 murine glioma cells were injected subcutaneously into the right caudate nuclei of C57BL/6 (RRID: MGI:2159769) male mice, specifically, using the coordinates as 0.6 mm AP, −2.0 mm ML, −3.2 mm DV at a rate of 0.25 μL/min using the stereotactic apparatus (RWD, China). The mice were divided into the following groups randomly: (1) sham group: mice underwent the same stereotaxic surgical procedures, with equivalent volume of PBS injected into the target site, (2) FUOM-sh-c treatment group: mice received subcutaneous injection of 10 μL GL261 cells transfected with FUOM-sh-c alone, (3) FUOM-oe treatment group: mice received subcutaneous injection of 10 μL GL261 cells transfected with FUOM-oe plasmids alone, (4) FUOM-sh-c combined CXCL13-oe treatment group: mice received subcutaneous injection of 10 μL GL261 cells transfected with FUOM-sh-c and CXCL13-oe plasmids, and (5) CXCL13-oe treatment group: mice received subcutaneous injection of 10 μL GL261 cells transfected with CXCL13-oe plasmid alone (mouse *n* = 8, respectively).

Animals were monitored daily to ensure humane endpoints, with animals displaying a protruded skull, hunched posture, extreme lethargy, or weight loss as cause for euthanasia. Long-term survival was defined at 30 days post tumor implantation, and the tumor-bearing brains were collected for histological analysis. Multiplex IHC was performed after serial sections of the tumor tissues. All animal experiments were conducted in accordance with the ethical guidelines of the Animal Research Committee of Nantong University Medical School (2022-018).

#### Quantitative real-time PCR (qRT-PCR)

Total RNA was isolated using RNA-easy Isolation Reagent (B2203HAA, Vazyme, SH, China), following the manufacturer’s protocol. Primer sequences were obtained and synthesized by Origene (https://www.origene.com.cn/; date of last access: 2025-10-11) and Tsingke Biotechnology (BJ, China). cDNA synthesis was performed using Hiscript III RT SuperMix for qPCR (+gDNA wiper) (R323-01, Vazyme, SH, China). Quantitative PCR was conducted with AceQ Universal SYBR qPCR Master Mix (Q511-02, Vazyme, SH, China), using the following cycling conditions: initial denaturation at 95 °C for 5 s, annealing at 60 °C for 15 s and extension at 72 °C for 15 s, for a total of 45 cycles. All assays were performed in triplicate. Gene expression levels were normalized to the cycle threshold of GAPDH, measured concurrently on the same plate. Relative expression levels were calculated using the 2-ΔΔCt method. The primer sequences used were shown in Supplemental Table 2.

#### Western blot assay

Proteins were extracted from glioma tissues and cell samples using RIPA lysis buffer (P0013B, Beyotime, SH, China), supplemented with protease and phosphatase inhibitors (P1260, Solarbio, BJ, China). Lysates from glioma and macrophage cells were centrifuged at 12,000×*g* for 15 min at 4 °C, and the supernatants were collected as conditioned media (CMs). Protein concentrations were determined using a BCA Protein Assay Kit (P0010, Beyotime, SH, China). Samples were prepared by mixing equal volumes of total protein with 2× loading buffer (P0015B, Beyotime, SH, China) and heating at 95 °C for 5 min. Then, proteins were separated on 7.5-12.5% SDS-PAGE gels (PG111, PG112, PG113, Epizyme Biotech, SH, China) and transferred onto PVDF membranes (0.22 μm or 0.44 μm pore size, ISEQ00010, IPVH00010, Millipore, Darmstadt, Germany). Membranes were blocked with 5% bovine serum albumin (BSA) in TBS for 2 h at room temperature to reduce non-specific binding. The membranes were incubated overnight with primary antibodies: anti-FUOM (1:200, ab188923, Abcam, Cambridge, UK, RRID: AB_2050251), anti-iNOS (1:1000, BA0326, Boster Bio, WH, China, RRID: AB_3081084), anti-CD163 (1:1000, 93498, CST, MIA, USA, RRID: AB_2800204), anti-CXCL13 (1:1000, MBA801-100, R&D systems, MN, USA, RRID: AB_2086049), anti-CD86 (1:800, orb388891, Biorbyt, Cambridge, UK, RRID: AB_2797422), and anti-GAPDH (1:10000, 60004-1-Ig, Proteintech, WH, China, RRID: AB_3677459), which were then incubated using peroxidase-conjugated secondary antibodies (Goat Anti-Rabbit IgG (H + L), 111-035-003; Goat Anti-Mouse IgG (H + L), 115-035-003, Jackson Immunoresearch, PA, USA) for 1.5–2 h at room temperature. Protein bands were visualized using an ECL chemiluminescence reagent (BL520A, Biosharp, BJ, China) and captured with a Chemiluminescent Imaging System (Tanon 4600 SF, SH, China). Band intensities were quantified using Gel-Pro Analyzer software (Version 4.0.00.001; Media Cybernetics, LP, China).

#### Co-immunoprecipitation (Co-IP)

HEK 293T cells were transfected with FUOM-oe and CXCL13-oe plasmids, respectively. After 48 h, the cells were lysed in NP-40 lysis buffer (P0013F, Beyotime, SH, China) supplemented with PMSF. The protein lysates were incubated overnight at 4 °C with protein A/G agarose beads (abs90323, Absin, SH, China) and either FUOM or CXCL13 antibodies. Normal immunoglobulin G (IgG, 98136-1-RR, Proteintech, WH, China; RRID: AB_3672282) was used as a negative control. The immune complexes were thoroughly washed, eluted, and subjected to SDS-PAGE, followed by immunoblotting with CXCL13 and FUOM antibodies.

#### CCK-8 assay

Cells in 96-well plates were seeded at 2 × 10^3^ per well in 100 μL of complete medium and 10 μL of CCK-8 reagent (B2282CAA, Vazyme, SH, China) for 2 h each day after 24 h of culture. Each sample was evaluated in triplicate. The absorbance of each pore at 450 nm was measured at 0, 24, 48, 72, and 96 h using a multimode microplate reader (Varioskan™ LUX, ThermoFisher, CA, USA). The cell growth curves were drawn to compare the proliferation ability of different groups of glioma cells.

#### Transwell assay

Transwell assays were performed to analyze the migration and invasion abilities of the glioma cells. Briefly, 5 × 10^4^ glioma cells were seeded in the upper layer coated with Matrigel (for cell invasion analysis) or left uncoated (for cell migration analysis) with serum-free medium, while complete DMEM/MEM medium with 10% FBS was placed in the lower chamber to induce cell invasion and/or migration. After 24–48 h of incubation, the migrating and/or invading cells were fixed using 4% PFA (#158172, paraformaldehyde, Sigma, CT, USA) and stained with 0.1% crystalline violet (C0121, Beyotime, SH, China) for 20 min. Quantitative analysis was performed using a light microscope.

#### Colony-forming assay

For the colony formation assay, we seeded 1 × 10^3^ transfected cells into each well of 6-well plates and cultured them in a complete medium supplemented with 10% FBS. After 14 days, we fixed the colonies with 4% PFA for 20 min and stained them with 0.01% crystal violet for 30 min to assess the colony-forming ability of the cells. Colonies with diameters exceeding 1 mm were then counted.

#### Human chemokine antibody microarray

To assess cytokine signaling related to macrophage functions following FUOM downregulation in T98G and U251 glioma cell lines, we used the RayBiotech Human Cytokine Antibody Array G series 2000 (ah-che-G1-8, Raybiotech, Norcross, GA). This array detects 44 macrophage-related cytokines. Serum samples, collected 48 h after transfection with sh-FUOM-c or FUOM-nc plasmids, were processed according to the manufacturer’s guidelines. The array was incubated with serum diluted in a 1:2 ratio for 2 h. Each sample was analyzed in duplicate. Following incubation, the slides were washed and incubated for 2 h with a biotinylated anti-cytokine antibody mixture. After a subsequent wash, development was performed using Cy3-conjugated streptavidin for 2 h. Signal detection was achieved with an Axon GenePix 4000B scanner (Molecular Devices, CA, USA), and the data were analyzed using GenePix Pro 6.0 software. Normalization was done using the negative controls on the array.

#### Enzyme-linked immunosorbent assay [25]

To quantify cytokine expression levels in glioma cell supernatants, we utilized an indirect ELISA assay performed in a sandwich format, as previously reported [26]. Supernatants from glioma cell lines transfected with sh-FUOM-c and FUOM-oe plasmids were coated onto microplates, and CXCL13 secretion was detected using a CXCL13-specific antibody (MBA801-100, R&D Systems, MN, USA) post-transfection. To adjust for potential variations in cytokine secretion, CXCL13 protein expression was normalized against the total protein content. For detection, HRP-conjugated Affinipure Goat Anti-Rabbit IgG (H + L) secondary antibodies (Proteintech, WH, China) were used following the manufacturer’s instructions. Chemiluminescent signals were measured using a Varioskan LUX Multimode Microplate Reader (Version 6.0.2, Thermo Scientific, CA, USA).

#### Macrophage polarization

THP-1 and Raw 264.7 macrophage cell lines were grown between passages 1–12 to block gene loss and impaired macrophage immune function using RPMI1640/DMEM with 10% FBS, 1% penicillin and streptomycin (C125C5, NCM biotech, Soochow, China) and 0.05 mM β-mercaptoethanol (BME, #21985023, Gibco, ThermoFisher, CA, USA) in a humidified environment containing 5% CO_2_ at 37 °C. For macrophage differentiation/activation, monocytes/macrophages (1 × 10^6^ cells/well) were seeded into 6-well plates with 50 nM PMA/phorbol 12-myristate 13-acetate (HY-18739, edChem Express, MO, USA) for 48 h in serum-free medium. Subsequently, the derived M0 macrophages were further activated by LPS (100 ng/mL, L2630, Sigma, DE) + INF-γ (20 ng/mL, AF-300-02, Peprotech, USA) or IL-4 (30 ng/mL, 200-04-5ug, Peprotech, USA) + IL-13 (30 ng/mL, 200-13-2ug, Peprotech, USA) for 48 h, polarized into M1- or M2-like macrophages respectively.

#### Preparation and culture of mouse bone marrow-derived macrophages (BMDMs)

Bone marrow cells were isolated from the femurs and tibias of mice and differentiated into bone marrow-derived macrophages (BMDMs) using M-CSF(HY-P7085, MCE, SH, China). Briefly, mice were euthanized, and the excess skin was removed to expose the lower limbs. The surrounding muscles of the femur, tibia, and fibula were carefully dissected to expose the bones. Bone marrow was flushed out from one end of each bone using a 23 G needle with 2–3 mL of PBS per bone, and the suspension was collected into a 50 mL centrifuge tube kept on ice. The bone marrow suspension was centrifuged at 200×*g* for 15 min at 4 °C. The resulting cell pellet was filtered and resuspended in DMEM supplemented with 10% heat-inactivated FBS, 1% penicillin-streptomycin, and M-CSF, and cultured for 4 days for macrophage differentiation. From day 4 onward, half of the macrophage culture medium was replaced with fresh medium containing the indicated stimulants to polarize BMDMs into M1- or M2-like macrophages for subsequent functional assays.

#### Flow cytometry analysis

Mouse BMDMs were resuspended in 1 mL FACS buffer for cell counting. Unstained BMDMs were used as negative controls. The cell suspensions were stained with anti-CD115-APC (Proteintech, APC-65095, WH, China; RRID: AB_3716392) and FITC Rat anti-Mouse F4/80 (Absin, abs1850175, SH, China; RRID: AB_3716393) antibodies to detect macrophage surface markers, along with a Live/Dead exclusion dye (Fixable Viability Stain 510, 564406, BD Horizon, USA; RRID: AB_2869572). Stained samples were analyzed using a Beckman CytoFLEX Flow Cytometer (Beckman, CA, USA) equipped with CytExpert software, and data were processed using FlowJo. Dead cells, debris, and doublets were excluded based on forward scatter (FSC) and side scatter (SSC) parameters in combination with the Live/Dead dye. Live single cells expressing both CD115 and F4/80 were identified as macrophages. Flow cytometric analysis indicated that approximately 83.29% of the cells were positive for CD115 and F4/80, confirming successful macrophage differentiation.

#### Conditioned medium (CM)

To establish an in vitro co-culture system between glioma cells and THP-1/BMDM-derived M1- or M2-like macrophages, 2 × 10^5^ glioma cells were seeded into six-well plates. For comparison, 2 × 10^5^ M1- or M2-like macrophages were also seeded. After 48 h incubation, the cells were collected for RNA extraction, and the supernatant was collected for co-cultured conditioned mediums (Co-CMs). All the Co-CMs were then centrifuged at 1500×*g* for 15 min to remove cell debris and used immediately. THP-1/BMDM-derived macrophages were polarized into M1- and M2-like macrophages as target cells. Lastly, Co-CMs were added to the medium of the target glioma cells at a ratio of 1:1.

#### Macrophage chemotaxis migration assay

A 24-well cell migration assay was performed as previously described [27]. Briefly, 400 μL of culture medium containing glioma CMs or control medium was added to the bottom chamber. THP-1 and BMDM-derived M1- or M2-like macrophages were suspended in a serum-free medium and added to the upper chamber. After incubation at 37 °C for 24 h, cells on the upper surface were removed, then fixed using 4% paraformaldehyde and stained with 0.1% crystalline violet for 20 min. Quantitative analysis was performed using a light microscope.

#### Macrophage phagocytosis assay

BMDM cells were resuspended at a density of 2 × 10⁶ cells per well and cultured in media from FUOM-sh-c/oe and control plasmid-treated supernatants. Latex beads conjugated with rabbit IgG-FITC (Phagocytosis Assay Kit–IgG FITC, No. 500290, Cayman Chemical, MI, USA) were added directly to pre-warmed culture medium at a final dilution of 1:1000. Cells were then incubated at 37 °C for 1-4 h to allow phagocytosis. After incubation, cells were gently washed three times with PBS to remove unbound beads. The uptake of fluorescent beads by macrophages was visualized under a fluorescence microscope after nuclear counterstaining.

#### Statistical analysis

The optimal cutoff for FUOM protein expression (39; range 0–100) was determined using X-tile (v3.6.1; Rimm Lab, Yale School of Medicine, CT, USA). Survival analysis was performed using the Kaplan–Meier method with the log-rank test in SPSS (v22.0; IBM Corp., NY, USA, RRID:SCR_002865). The age cutoff of 60 years was determined based on a previous retrospective analysis that evaluated the impact of age on outcomes in transplanted myeloma patients (≤66 years) [28]. Analyses were conducted using GraphPad Prism software (www.graphpad.com, last date of access: 2025-10-11, Version 9.0.0; CA, USA). Two-group comparisons were assessed using unpaired or paired two-tailed Student’s *t*-tests, and ANOVA was applied for multiple groups. All quantitative data were expressed as mean ± SD or mean ± 95% CI, and *P* < 0.05 was considered statistically significant.

### Ethics approval and patient consent to participate

All patients provided written informed consent before sampling, after receiving comprehensive information about the purpose, procedures, potential benefits, and risks associated with the collection and use of their specimens. The original written informed consent forms are archived in the Clinical Biobank of the Affiliated Hospital of Nantong University. All procedures performed in this study involving human participants were in accordance with the ethical standards of the Human Research Ethics Committee of the Affiliated Hospital of Nantong University (Grant No. 2018-K020) and with the 1964 Helsinki Declaration and its later amendments or comparable ethical standards. All animal experiments were conducted in accordance with the ethical guidelines of the Animal Research Committee of Nantong University Medical School.

## Results

### FUOM was upregulated in glioma tissues and related to patient prognosis

**Fig. 1:**
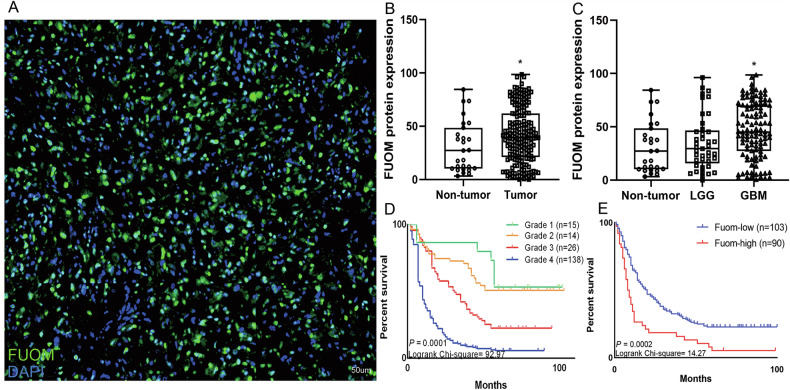
FUOM protein expression related to glioma patient prognosis. **A** Image of FUOM protein expression on glioma TMAs (Scale bar = 50 µm). **B**, **C** Quantified TMA-mIHC results showing FUOM expression between the non-tumor brain, LGG, and GBM patients (*P < 0.05). **D** Kaplan–Meier survival estimates between Grade I–Grade IV glioma among clinical patient cohort (*n* = 193). **E** Kaplan–Meier survival estimates between high and low FUOM expression among the glioma patient cohort (*n* = 193).

To investigate the role of FUOM in glioma initiation and progression, we first examined its expression using TMA-mIHC staining. FUOM protein was predominantly localized in the cytoplasm of glioma cells (Fig. 1A). Moreover, FUOM protein expression was significantly higher in glioma tissues compared to non-tumor brain tissues, with the highest levels observed in GBM patients (Fig. 1B, C, Supplemental Fig. 1A–C and A1–C1). ROC curve indicated that FUOM had strong predictive performance for overall survival, with an AUC of 0.873 (95% CI: 0.856–0.890, Supplemental Fig. 1D). Furthermore, transcriptional upregulation of FUOM mRNA in glioma was associated with poorer patient prognosis in the CGGA database (HR: 2.43, *P* < 0.001, Supplemental Fig. 1E, F) and TCGA & GTEx database (HR: 1.91, *P* < 0.001, Supplemental Fig. 1G–I).

In our glioma patient cohort, patients were further categorized into low/no and high FUOM expression groups based on an optimized cutoff. FUOM expression was significantly associated with glioma WHO grade (χ^2^ = 12.876, *P* = 0.004) and histological type (χ^2^ = 9.860, *P* = 0.043) (Table 1). Subsequent univariate and multivariate Cox regression analysis identified FUOM expression (HR = 1.980, *P* = 0.01), age (HR = 1.669, *P* = 0.002), and glioma grade (HR = 1.316, *P* = 0.001) as independent predictors of overall survival (Table 2). Kaplan–Meier analysis confirmed that high FUOM expression and higher glioma grade correlated with shorter patient OS (Fig. 1D, E), establishing FUOM as an independent prognostic marker of poor outcome in patients with glioma.

**Table 1:** Relationship between the expression of FUOM and clinicopathological characteristics in glioma patients.

Characteristic	*n*	Low or no expression (%)	High expression (%)	Pearson χ^2^	*P*-value
Total	193	103 (53.37)	90 (46.33)		
Gender				0.106	0.689
Male	113	58 (51.33)	55 (48.67)		
Female	80	45 (56.25)	35 (43.75)		
Age				2.06	0.151
<60	124	70 (56.45)	54 (43.55)		
≥60	69	33 (47.82)	36 (52.18)		
Histological type				9.860	0.043*
a	120	56 (46.67)	64 (53.33)		
b	29	22 (75.86)	7 (24.14)		
c	33	19 (57.58)	14 (42.42)		
d	11	6 (54.55)	5 (45.45)		
Molecular type				0.049	0.824
IDH1 ^R132H^ mut	176	93 (52.84)	83 (47.16)		
IDH1 ^R132H^ WT	17	10 (58.82)	7 (41.18)		
WHO grade				12.876	0.004*
1 and 2	29	22 (75.86)	7 (24.14)		
3	26	17 (65.38)	9 (34.62)		
4	138	64 (46.38)	74 (53.62)		
Chemotherapy				0.460	0.498
Yes (TMZ)	24	11 (45.83)	13 (51.17)		
None	169	92 (54.44)	77 (45.56)		
Radiotherapy				0.907	0.635
Yes	38	22 (57.89)	16 (42.11)		
No	155	81 (52.26)	74 (47.42)		

a: Astrocytoma, IDH1^R132H^ mutant.

b: Oligodendroglioma, IDH1^R132H^ mutant.

c: GBM, IDH1^R132H^ wildtype.

d: Pilocytic astrocytoma, pilomyxoid astrocytoma, and pleomorphic xanthoastrocytoma.

*P < 0.05.

**Table 2:** Univariate and multivariable analysis of prognostic factors for 5-year survival in glioma patients.

	Univariate analysis					Multivariate analysis		
	HR	*P* < |z|	95% CI		HR	*P* < |z|	95% CI	
FUOM expression High vs low/no	1.980	0.0001*	1.356	2.892	1.568	0.025*	1.059	2.323
FUOM^+^CXCL13^+^ expression High vs low/no	1.832	0.0001*	1.776	1.839	1.844	0.0001*	1.785	1.907
Age (years) ≤60 vs >60	1.961	0.0001*	1.408	2.732	1.69	0.046*	1.483	1.984
Gender Male vs female	1.519	0.014*	1.088	2.120	1.383	0.04*	0.983	1.946
Histological type a vs b vs c vs d	0.919	**0.197**	0.809	1.045				
Molecular type IDH1^R132H^/mut vs IDH1 ^R132H^/WT	0.874	0.667	0.472	1.616				
WHO grade 1 & 2 vs 3 vs 4	1.385	0.0001*	1.169	1.641	1.316	0.002*	1.112	1.557
Chemotherapy TMZ vs none	0.749	0.250	0.457	1.226				
Radiotherapy Yes vs no	0.721	0.079	0.501	1.038				

a: Astrocytoma, IDH1^R132H^ mutant.

b: Oligodendroglioma, IDH1^R132H^ mutant.

c: GBM, IDH1^R132H^ wildtype.

d: Pilocytic astrocytoma, pilomyxoid astrocytoma, and pleomorphic xanthoastrocytoma.

*P < 0.05.

### FUOM promoted glioma cell growth in vitro

**Fig. 2:**
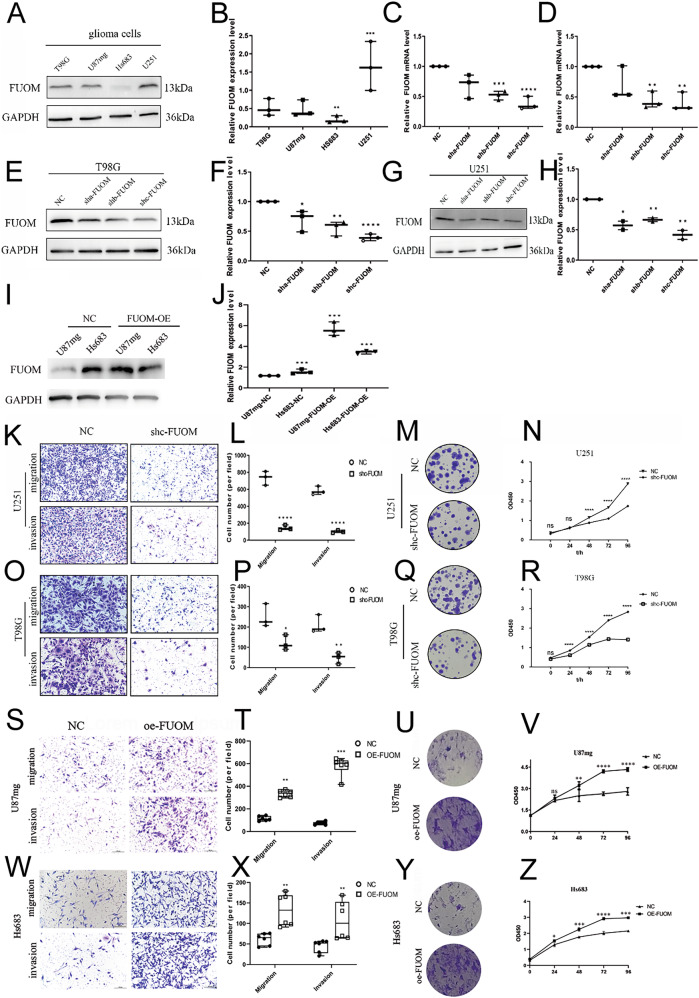
FUOM was indispensable for primary glioma cell progression. **A**, **B** Quantification analysis of FUOM expression on four glioma cell lines (**P < 0.01, ***P < 0.005). **C**, **D** Quantification analysis of FUOM mRNA expression on T98G and U251 glioma cell lines after FUOM downregulation using sh-FUOM-c plasmid (***P < 0.005, ****P < 0.0001). **E**, **F** Quantification analysis of FUOM protein expression on T98G glioma cell line after FUOM downregulation using sh-FUOM-c plasmid (*P < 0.05, **P < 0.01, ****P < 0.0001). **G**, **H** Quantification analysis of FUOM protein expression on U251 glioma cell line after FUOM downregulation using plasmids (*P < 0.05, **P < 0.01). **I**, **J** Quantification analysis of FUOM protein expression on U87mg, Hs683, FUOM-OE-U87mg and FUOM-OE-Hs683 glioma cell lines after FUOM overexpression using FUOM-oe plasmid (***P < 0.005). **K**, **L** Quantification analysis of the migration and invasion abilities of U251 glioma cell line after FUOM downregulation (****P < 0.0001). **M** Colony-forming assay of U251 glioma cell line after FUOM downregulation. **N** CCK-8 assay of U251 glioma cell line after FUOM downregulation (****P < 0.0001). **O**, **P** Quantification analysis of the migration and invasion abilities of T98G glioma cell line (*P < 0.05, **P < 0.01). **Q** Colony-forming assay of U251 glioma cell line after FUOM downregulation. **R** CCK-8 assay of U251 glioma cell line after FUOM downregulation (****P < 0.0001). **S**, **T** Quantification analysis of the migration and invasion abilities of U87mg glioma cell line after FUOM overexpression (**P < 0.01, ***P < 0.005). **U** Colony-forming assay of U87mg glioma cell line after FUOM overexpression. **V** CCK-8 assay of U87mg glioma cell line after FUOM overexpression. **W**, **X** Quantification analysis of the migration and invasion abilities of Hs683 glioma cell line after FUOM overexpression (**P < 0.01). **Y** Colony-forming assay of Hs683 glioma cell line after FUOM overexpression. **Z** CCK-8 assay of Hs683 glioma cell line after FUOM overexpression (*P < 0.05, ***P < 0.005, ****P < 0.0001).

To investigate the role of FUOM protein in glioma development, we first assessed its expression in four glioma cell lines (U251, U87mg, T98G, and Hs683). FUOM protein expression levels were detected as higher in U251 and T98G, but lower in U87mg and Hs683 glioma cell lines (Fig. 2A, B). Thus, we manipulated *FUOM* gene expression by knocking down *FUOM* in U251 and T98G cells and overexpressing it in U87mg and Hs683 cells. *FUOM* knockdown effectively reduced FUOM mRNA and protein expression levels (Fig. 2C–H), whereas overexpression of *FUOM* significantly increased its protein expression levels compared to FUOM-nc controls (Fig. 2I, J). Moreover, functional assays demonstrated that *FUOM* depletion impaired glioma cell viability, proliferation, migration, and invasion in U251 and T98G cell lines (Fig. 2K–R), while *FUOM* overexpression enhanced these functions in U87mg and Hs683 cell lines (Fig. 2S–Z). Taken together, these results suggest that FUOM is a key regulator of glioma tumorigenesis and progression in vitro.

### FUOM facilitated M2-like macrophage infiltration in glioma patients

**Fig. 3:**
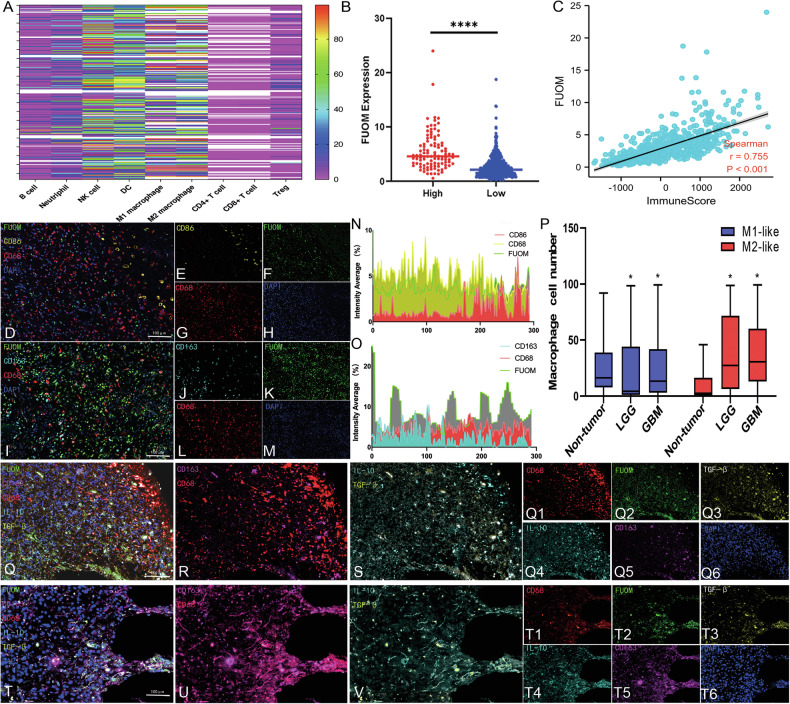
FUOM maintained appropriate cytokine release of CXCL13 in glioma cells. **A** Heatmap analysis of the correlations between FUOM protein expression and infiltrated immune cells. **B** The distribution of high and low FUOM expression on the clinical patient cohort (*n* = 193, ****P < 0.0001). **C** Correlation between FUOM expression and immune scores of macrophages (r = 0.755, *P* < 0.001). **D**–**H** Representative images showing FUOM and M1-like macrophage (CD68^+^ and CD86^+^) immunofluorescence in grade IV glioma TMA (Scale bar = 100 µm). **I**–**M** Representative images show FUOM and M2-like macrophage (CD68^+^ and CD163^+^) immunofluorescence in grade IV glioma TMA (Scale bar = 100 µm). **N**, **O** Quantitative analysis of M1- and M2-like macrophage fluorescence intensity, together with their co-expression correlation with FUOM expression. **P** The quantified cell numbers of M1- and M2-like macrophages in the glioma TME that correlated with FUOM expression (*P < 0.05). **Q**–**S** Representative immunofluorescence images showing IL10 and TGF-β within high-FUOM expression and M2-like macrophage (CD68^+^ and CD163^+^) regions in glioma TMA (Scale bar = 100 µm). **T**–**V** Representative immunofluorescence images showing IL10 and TGF-β within low-FUOM expression and M2-like macrophage (CD68^+^ and CD163^+^) regions in glioma TMA (Scale bar = 100 µm).

Recent evidence indicates that tumor cells recruit immune cell infiltration to sustain glioma pathogenesis and drive malignant progression [13]. To explore the involvement of FUOM in glioma immune microenvironment, we first evaluated the correlations between various infiltrated immune cell types and FUOM protein expression on patient TMAs (Fig. 3A). Among the 9 types of immune cells analyzed, M1/M2-like macrophages, dendritic cells (DCs), natural killer (NK) cells, and neutrophils displayed significant associations with FUOM expression (Fig. 3A). Based on the calculated immune infiltration scores, patients were divided into low/no and high immune infiltration groups. *FUOM* mRNA expression levels were significantly higher in the high immune infiltration group (Fig. 3B), and high *FUOM* mRNA expression showed a positive correlation with macrophage infiltration among these 9 immune cell types (r = 0.755, Fig. 3C). To further delineate the macrophage infiltration landscape and its correlation with FUOM in the glioma TME, TMA-mIHC was performed. M1-like macrophages were identified using anti-CD86 and anti-CD68 antibodies (M1 panel, Fig. 3D–H), while M2-like macrophages were labeled with anti-CD163 and anti-CD68 antibodies (M2 panel, Fig. 3I–M). FUOM staining was stronger and clustered near glioma cells in the M2 panel, showing spatial proximity to CD163⁺/CD68⁺ M2-like macrophages (Fig. 3I, M). Fluorescence co-localization analysis indicated that FUOM expression was more consistent with M2-like macrophages in both localization and intensity compared to M1-like macrophages (Fig. 3N, O). Moreover, quantification analysis revealed M2-like macrophage infiltration exhibit vast difference between the non-tumor brain and GBM brain, whereas M1-like macrophage showed less difference. Specifically, M2-like macrophage number was much less in the non-tumor brain, but increased significantly in GBM (Fig. 3P). Consistently, regions with high FUOM expression exhibited elevated levels of functional cytokines IL-10 and TGF-β within the M2-like macrophage population, indicating active engagement of myeloid immune signaling networks (Fig. 3Q–S). In contrast, areas with low FUOM expression showed relatively low M2-like macrophage infiltration and correspondingly low cytokine expression (Fig. 3T–V). This suggests that M2-like macrophage enrichment is closely associated with enhanced immune cytokine activity within the glioma TME. Collectively, these findings suggest that FUOM promotes M2-like macrophage infiltration in glioma TME.

### FUOM maintained appropriate cytokine release of CXCL13 in glioma cells

**Fig. 4:**
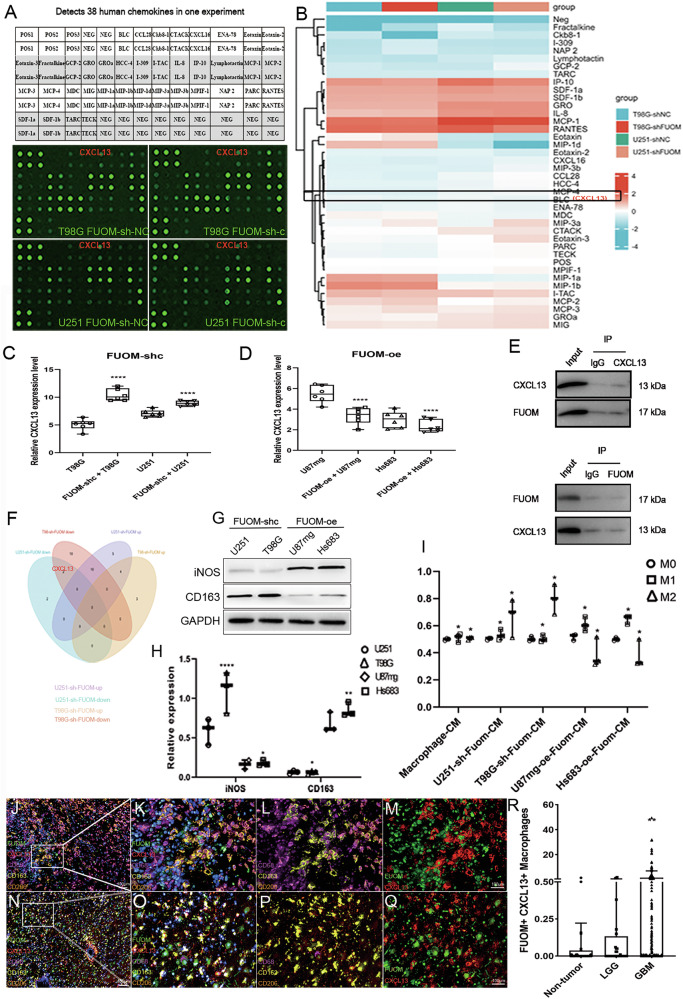
CXCL13 activated M2-like macrophage polarization in the TME. **A** Human cytokine antibody array showing 44 macrophage-related cytokine expression in the super-serum of T98G and U251 cell lines upon FUOM downregulation. **B** Heatmap analysis of cytokine expression in the super-serum of T98G and U251 cell lines upon FUOM downregulation. **C**, **D** Relative CXCL13 expression in FUOM-sh-c/oe treated glioma cells using Elisa (****P < 0.0001). **E** Identification of a physical interaction between FUOM and CXCL13 in HEK 293T cells by Co-IP assays. **F** Venn’s analysis shows CXCL13 was upregulated after FUOM downregulation on T98G and U251 glioma cell lines. **G**, **H** Protein expression of surface markers iNOS and CD163 on THP-1 induced M0, M1, and M2 macrophages. **I** Quantified results of THP-1 induced M1 and M2 macrophage marker expression (*P < 0.05, **P < 0.01, ****P < 0.0001). **J**–**M** Representative image (**G**) and magnified region (**H**) showing distribution and expression of M2-like macrophage (**I**), FUOM, and CXCL13 (**J**) in glioma (grade IV) tissue (Scale bar = 100 µm). **N**–**Q** Representative image (**K**) and magnified region (**L**) showing distribution and expression of M2-like macrophage (**M**), FUOM, and CXCL13 (**N**) in non-tumor brain tissue (Scale bar = 100 µm). **R** Quantitative analysis of FUOM^+^ CXCL13^+^ macrophages in non-tumor, LGG and GBM patient TMAs (*P < 0.05).

To clarify how FUOM facilitates macrophage infiltration, a human cytokine antibody array was used to profile 44 macrophage-related cytokine release in the conditioned co-culture media of T98G and U251 glioma cells after FUOM knockdown (Fig. 4A). Downregulation of FUOM in T98G and U251 glioma cell lines markedly increased the CXCL13 expression (BLC, BCA-1, Fig. 4B). Quantitative analysis of released cytokine levels in the culture supernatants further confirmed a negative feedback regulation between FUOM expression and CXCL13 secretion, as illustrated in the heatmap (Fig. 4B). Consistently, ELISA assays of Co-CMs from FUOM-sh-c and FUOM-oe glioma cells revealed that FUOM knockdown significantly upregulated CXCL13, whereas FUOM overexpression suppressed CXCL13 secretion (Fig. 4C, D. ****P < 0.0001). To explore their potential interaction, co-immunoprecipitation (Co-IP) assays were conducted in HEK293T cells by overexpressing FUOM or CXCL13, and the results clearly demonstrated a direct exogenous interaction between FUOM and CXCL13 (Fig. 4E). Venn’s analysis together shows CXCL13 was upregulated after FUOM downregulation on T98G and U251 glioma cell lines (Fig. 4F). To further determine which macrophage subtype responds to FUOM-CXCL13 signaling, we assessed macrophage surface marker expression using co-culture systems. Specifically, the M1/M2 activation states of THP-1 macrophages were examined upon co-culturing with FUOM-sh-c and FUOM-oe treated glioma cells. Notably, the M2-like macrophage marker CD163 was downregulated on FUOM-sh-c group. In contrast, M1-like macrophage marker iNOS was upregulated in the FUOM-oe group (Fig. 4G, H). Moreover, the supernatants from the Co-CMs were further analyzed. Consistently, M2-like macrophages were preferentially activated in the FUOM-sh-c Co-CMs group, indicating that FUOM downregulation promotes CXCL13-mediated M2-like macrophage polarization in vitro (Fig. 4I).

Furthermore, mIHC was performed to localize M2-like macrophage populations (CD68^+^CD163^+^CD206^+^ myeloid cells) in relation to FUOM and CXCL13 expression. FUOM protein accumulated within and around the M2-like macrophages in glioma regions, while M2-like macrophages aggregated in FUOM-enriched areas (Fig. 4J–M). Similarly, CXCL13 was clustered in regions enriched with M2-like macrophages (Fig. 4N–Q). In contrast, non-tumor brain tissues exhibited a markedly lower density of M2-like macrophages (Fig. 4R). These results suggested that downregulated FUOM expression directly promotes CXCL13 secretion, thus activating M2-like macrophage polarization in glioma. Importantly, we used functional rescue experiments to highlight FUOM as a key regulator of glioma progression in vitro. First, we treated FUOM knockdown glioma cells with full-length His-tagged FUOM protein (Isoform-1) and confirmed its efficacy in restoring glioma cell proliferation in vitro (Supplemental Fig. 2A, B). Meanwhile, we overexpressed CXCL13 in FUOM-sh-c-treated glioma cells, which indicated further impaired glioma cell proliferation, migration, and invasion function, especially in U251 glioma cell lines (Supplemental Fig. 2D–I).

As previously described, the CXCL13-CXCR5 axis activates intracellular signaling through the canonical GPCR cascade, engaging downstream effectors such as nuclear factor-κB (NF-κB), protein kinase B (Akt), extracellular signal-regulated kinase (ERK), and p38 mitogen-activated protein kinase (p38 MAPK). Thus, we hypothesized that differential FUOM expression in glioma cells might trigger specific regulators within these pathways, thereby modulating CXCL13 secretion. To dissect the underlying mechanism, we applied pathway inhibitors targeting PI3K/AKT (LY294002), ERK (PD98059), and MAPK (Rac1/Rac2 inhibitor, EHT1864) in FUOM-downregulated glioma cell lines. Notably, PI-3K inhibition markedly suppressed CXCL13 expression (Supplemental Fig. 2C) and elicited a marked activation of downstream effectors, including PI-3KCa and PI-3KR1, which were upregulated upon inhibitor treatment but suppressed following CXCL13 overexpression (Supplemental Fig. 2I, J). In contrast, RAC1, RAC2, and PTEN remained largely unaffected either by inhibitor or CXCL13 overexpression treatment (Supplemental Fig. 2K–M). Collectively, these findings provide additional mechanistic evidence linking the FUOM-CXCL13 axis in mediating M2-like macrophage function through activation of the PI3K/AKT signaling pathway, providing mechanistic support for its role in shaping the glioma immune microenvironment.

### CXCL13 activated M2-like macrophage polarization and migration in the TME

**Fig. 5:**
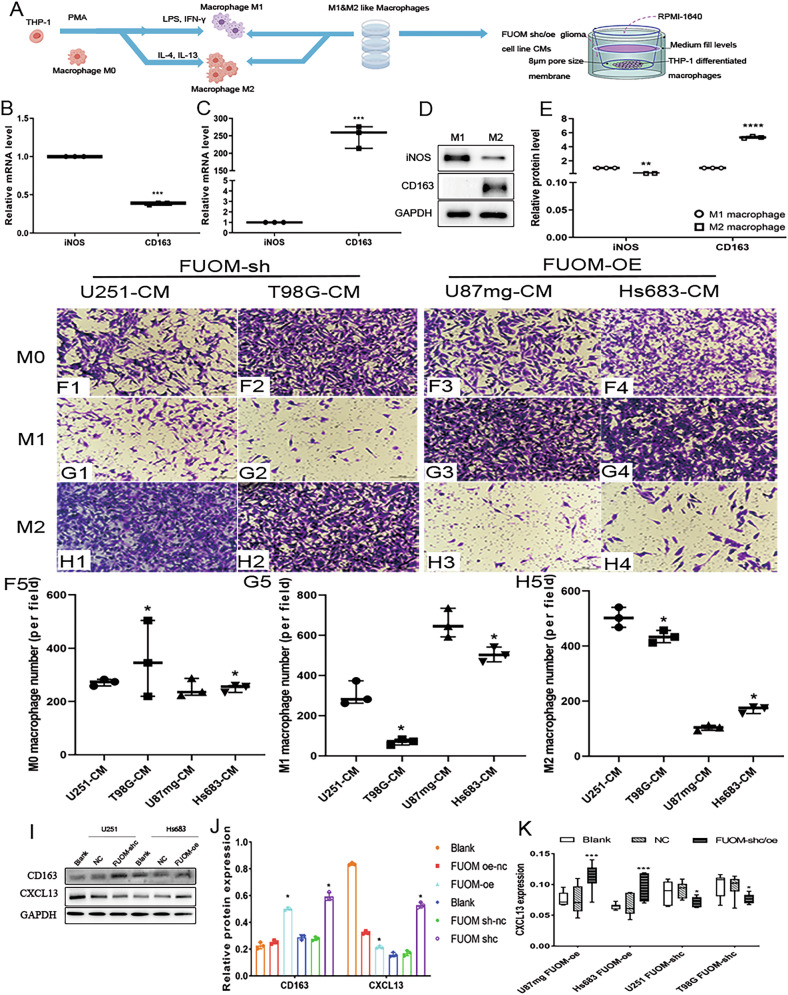
Loss of FUOM triggered an inflammatory response by macrophages. **A** Schematic diagram of the macrophage and glioma cell co-culture using condition media. **B**, **C** Relative iNOS and CD163 mRNA expression on THP-1 induced M1 and M2 macrophages. **D**, **E** Relative iNOS and CD163 protein expression on THP-1 induced M1 and M2 macrophages (**P < 0.01, ****P < 0.0001). **F1**–**F5** Representative images of the chemotaxis functions of CMs from U251 (**F1**), T98G (**F2**), U87mg (**F3**), Hs683 (**F4**) glioma cells toward M0-like macrophages and related quantification analysis (**F5**). **G1**–**G5** Representative images of the chemotaxis functions of CMs from U251 (**G1**), T98G (**G2**), U87mg (**G3**), and Hs683 (**G4**) glioma cells toward M1-like macrophages and related quantification analysis (**G5**). **H1**–**H5** Representative images of the chemotaxis functions of CMs from U251 (**H1**), T98G (**H2**), U87mg (**H3**), and Hs683 (**H4**) glioma cells toward M2-like macrophages and related quantification analysis (**H5**). **I**, **J** Relationship between CXCL13 and CD163 protein expression after up- and downregulation of FUOM on glioma cell lines. **K** CXCL13 expression on four glioma cell lines after up and downregulation of FUOM on glioma cell lines.

To elucidate the mechanisms by which CXCL13 promotes M2-like macrophage infiltration into glioma sites, thereby modulating glioma progression, we analyzed Co-CMs, which are known to reflect macrophage functional states (Fig. 5A). Our results showed that differentiation of THP-1 cells into M1-like macrophages markedly increased *iNOS* expression, whereas induction into M2-like macrophages significantly elevated *CD163* expression, as determined by qPCR. Consistently, western blot analysis further confirmed enhanced iNOS expression in M1-like macrophages and elevated CD163 levels in M2-like macrophages after 48 h of polarization (Fig. 5B–E). Additionally, we explored the chemotactic effects of CXCL13 secretion regulated by FUOM on M1- and M2-like macrophages using glioma cell-derived CMs in a transwell co-culture system, allowing indirect cell-cell contact (Fig. 5F–H). The polarization status of macrophages induced by different tumor cell CMs was further evaluated. The results showed that M2-like macrophages exhibited significantly greater migration ability toward CXCL13-enriched condition medium derived from FUOM knock-downed glioma cells compared with M0- and M1-like macrophages (*P* < 0.05, Fig. 5F5, G5, H5). Conversely, in FUOM-oe CMs, migration of M1 and M2-like macrophages was markedly reduced relative to FUOM-sh-c CMs (Fig. 5F5, G5, H5). To further evaluate the functional impact of CXCL13 on M2-like macrophages, we co-cultured FUOM-downregulated glioma cells with mouse bone marrow-derived macrophages (BMDMs) with/without CXCL13 treatment (Supplemental Fig. 3A). Flow cytometric analysis confirmed that approximately 83.29% of BMDMs were CD115⁺F4/80⁺ after 4 days of M-CSF stimulation (Supplemental Fig. 3B, C). To assess macrophage functionality, phagocytosis assays were performed on BMDMs, M1-BMDM, and M2-BMDM (Supplemental Fig. 3D–F). Then, we used a co-culture system to further demonstrate the role of CXCL13. Overexpression of CXCL13 on FUOM-downregulated glioma cells markedly enhanced the phagocytic capacity of M2-like BMDMs (Supplemental Fig. 3I, J), whereas its effect on M1-like BMDMs was relatively minimal (Supplemental Fig. 3G, H). Furthermore, CXCL13 overexpression in FUOM knockdown glioma cells (T98G and U251) further enhanced M2-like BMDM chemotactic activity (Supplemental Fig. 3K–P), supporting that FUOM regulates M2-like macrophage polarization and chemotaxis function through CXCL13 signaling.

Moreover, we observed an increase in the M2-like marker CD163 and a decrease in the M1-like marker iNOS in the co-cultured system (Fig. 5I, J). Furthermore, CXCL13 levels in the Co-CMs derived from M2-like macrophages co-cultured with glioma cell lines exhibiting altered FUOM expression were quantified using ELISA. The results showed that Co-CMs from M2-like macrophages displayed elevated CXCL13 levels when co-cultured with glioma cells in which FUOM was downregulated (Fig. 5K). Notably, inhibition of FUOM in glioma cells specifically increased CXCL13 secretion, thereby promoting M2-like macrophage activation and reshaping the tumor microenvironment toward a more immunologically active phenotype. Next, we investigated the regulatory mechanisms through which FUOM modulates macrophage recruitment and the subsequent secretion of macrophage-derived factors, given that alterations in FUOM expression may critically influence the crosstalk between glioma cells and myeloid immune cells. Our results showed that co-culture of M2-like macrophages with FUOM knocked-down U251 and T98G glioma cell lines led to decreased expression of IL-10 and TGF-β, both in THP-1-derived macrophage (Supplemental Fig. 4A, B) and M2-BMDMs (Supplemental Fig. 4C, D), indicating an enhanced inflammatory milieu associated with glioma progression. These findings were consistent with mIHC staining results showing increased IL-10 and TGF-β expression (Fig. 3Q–V).

To further identify the regulatory factors influencing macrophage polarization, BMDMs were co-cultured with FUOM-deficient glioma cells. The results revealed multiple alterations in macrophage regulatory molecules, particularly in M2-polarized BMDMs. Notably, when co-cultured with M2-BMDMs, IRF3 and STAT3 were both identified as key downstream regulators in the FUOM-CXCL13 signaling axis, favoring M2-like polarization, which were upregulated upon FUOM knockdown and reversed by CXCL13 overexpression, confirming their involvement in FUOM-mediated CXCL13 regulation (Supplemental Fig. 4F, G). Although both IRF3 and STAT3 contribute to M2-like macrophage differentiation, the marked accumulation of CXCL13 following FUOM knockdown may override IRF3-mediated regulation, leaving STAT3 as the principal upstream effector of CXCL13. In contrast, no significant activation was detected among M1 regulator NF-κB or the M2 regulator SMAD2 (Supplemental Fig. 4E, H). Collectively, FUOM suppression in glioma cells specifically upregulated CXCL13 through STAT3 activation, thereby promoting M2 macrophage polarization and contributing to tumor microenvironment remodeling.

### FUOM promotes in vivo macrophage recruitment and M2-like polarization to modulate antitumor immunity

**Fig. 6:**
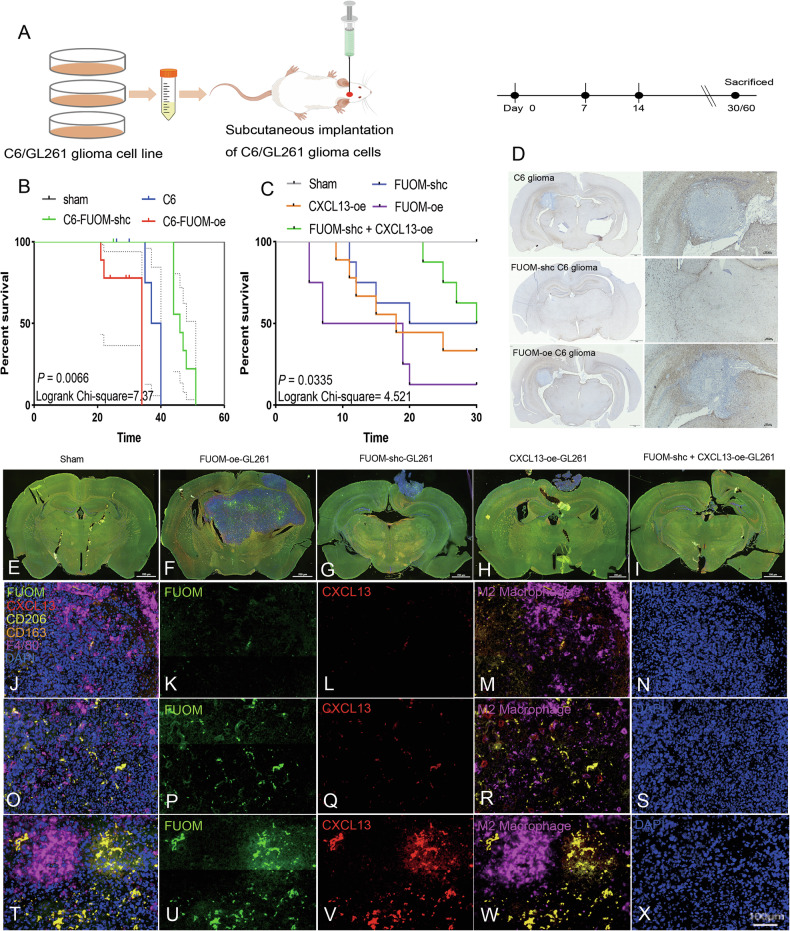
FUOM inhibition related to reduced glioma genesis in vivo. **A** Schematic illustration of the experimental procedures of glioma animal (rat / mouse) model construction in this study. **B** Kaplan–Meier survival curves of rats bearing subcutaneous C6 gliomas and their corresponding controls treated with FUOM-sh-c/oe treated C6 cells (*P* = 0.0066). The C6 implantation group served as control. **C** Kaplan–Meier survival curves of mice bearing subcutaneous GL261 gliomas treated with FUOM and/or CXCL13 and their corresponding controls (*P* = 0.0335). The sham group was used as control. **D** Representative images of DAB staining of GFAP (neighborhoods to tumor cells) showing brain tumors in rat C6 glioma models (Scale bar = 1 mm). **E**–**I** Representative images of mIHC staining of FUOM (green), CXCL13 (red), M2 macrophages (F4/80 orange, CD206 yellow, CD163 magenta) showing FUOM and/or CXCL13 treatment efficacy in mouse GL261 glioma models (Scale bar = 700 um). **J**–**N** Representative images of mIHC staining of the infiltrated F4/80^+^ CD163^+^ CD206^+^ M2-like macrophages in the sham group of the GL261 glioma-bearing mice (Scale bar = 100 µm). **O**–**S** Representative images of mIHC staining of the infiltrated F4/80^+^ CD163^+^ CD206^+^ M2-like macrophages in the FUOM-shc treatment group of the GL261 glioma-bearing mice (Scale bar = 100 µm). **T**–**X** Representative images of mIHC staining of the infiltrated F4/80^+^ CD163^+^ CD206^+^ M2-like macrophages in the FUOM-shc combined with CXCL13-oe treatment group of the GL261 glioma-bearing mice (Scale bar = 100 µm).

Since the expression of FUOM and CXCL13 was positively correlated with glioma patient outcomes (Fig. 1E), we next investigated whether in vivo administration of FUOM and/or CXCL13 could enhance antitumor efficacy and promote neurological recovery. To this end, we established primary glioma models by subcutaneously implanting C6 rat and GL261 mouse glioma cell lines (Fig. 6A). Glioma-bearing rats were maintained for up to 51 days (range: 24–51 days, median survival time: 33.83 ± 8.22 days), while glioma-bearing mice were kept for up to 30 days (range: 5–30 days, median survival time: 22.21 ± 8.96 days). All animals were sacrificed at their near-terminal stage (Fig. 6A, right). Kaplan–Meier survival analysis revealed that FUOM-sh-c-treated group exhibited significantly prolonged survival (Fig. 6B), even longer survival time was observed when combined with CXCL13 treatment (Fig. 6C). Moreover, the total glioma volume was markedly reduced following FUOM-sh-c treatment, while FUOM-oe treatment led to increased glioma cell infiltration (Fig. 6D). In addition, CXCL13-oe treatment alone produced a similar antitumor effect, and when combined with FUOM-sh-c treatment, glioma volume was almost eliminated in the mouse model (Fig. 6E–G).

In the sham group, M2-like macrophages were rarely observed within the glioma region, consistent with the expression patterns of FUOM and CXCL13 (Fig. 6J–N). In the FUOM-sh-c treated group, we detected a marked increase in M2-like macrophage expression and infiltration. These cells penetrated the extravascular stroma, traversing the vascular barrier to reach the inner tumor and tumor-associated parenchymal cells within the FUOM-enriched glioma TME (Fig. 6O–S). However, in FUOM-sh-c combined with CXCL13 treatment group, M2-like macrophages largely accumulated at the injection site with significantly reduced tumor size (Fig. 6T–X).

In conclusion, FUOM exhibits a potent anti-glioma effect through M2-like macrophages when combined with CXCL13 treatment. The in vivo FUOM-depleted glioma microenvironment appeared to feedback that modulated the behavior of infiltrating M2-like macrophages, potentially enhancing therapeutic efficacy and improving neurological outcomes.

## Discussion

In the present work, we investigated the role of glioma-specific FUOM expression in glioma progression and explore the underlying mechanisms governing the recruitment of TAMs, which represent the predominant immune cell population in glioma, comprising up to 50% of the immune cells within the tumor. We found that deficiency in FUOM upregulated CXCL13 expression, which in turn promoted macrophage activation and polarization toward an M2-like phenotype. This shift enhanced macrophage infiltration into the glioma TME and was associated with increased GBM aggressiveness. Collectively, our results identified FUOM as an independent prognostic indicator for glioma patient survival and highlighted its role as a specific mediator in the response of M2-like macrophages within the glioma TME.

Secretory proteins, which are widely distributed throughout tumor tissues and released into the bloodstream, play a pivotal role in cancer development and tumor cell migration [29]. These tumor cell-secreted proteins regulate cytokine release, facilitate communication between the tumor and immune cells, thereby influencing tumor growth and impacting the efficacy of cancer treatment in clinical settings. Consequently, secretory proteins have garnered increasing attention as valuable biomarkers and promising therapeutic targets across various cancer types [30]. In glioma, FUOM expression is markedly elevated in tumor tissues compared with non-tumorous brain tissues, serving as an independent prognostic indicator. Previous studies have identified CXCL13 as a key mediator of tumor-associated M2-like macrophage activity during malignant glioma progression, linking it to aggressive tumor behavior and poor patient outcomes [21]. In the present study, we demonstrated that FUOM expression in glioma is closely correlated with enhanced proliferation and invasion, primarily through the regulation of CXCL13. Elevated CXCL13 expression further promotes macrophage infiltration and polarization toward an M2-like phenotype, thereby fostering glioma progression, as evidenced by co-culture experiments. To counteract excessive FUOM secretion and prevent intracellular acidosis, glioma appear to upregulate the expression of CXC cytokines, particularly CXCL13. FUOM is detected both locally within the tumor extracellular matrix and systemically in patient serum. Consequently, high FUOM expression contributes to cancer progression by promoting cell proliferation, migration, and invasion. Taken together, these findings suggest that the FUOM-CXCL13-macrophage regulatory axis plays a crucial role in glioma progression and is associated with unfavorable clinical outcomes, highlighting its potential as a promising target for therapeutic intervention.

Comprehensive analyses of the cellular and molecular landscape of glioma TME have revealed diverse signaling networks and immune cell populations that collectively shape glioma TME and represent potential therapeutic targets. Also, immunotherapies have rapidly advanced and shown promising efficacy across multiple cancer types [31]. Among immune cell populations, monocyte-derived macrophages are notable for their capacity to infiltrate the brain and exhibit distinct functional and phenotypic states. These cells serve dual roles-supporting immune surveillance while facilitating tumorigenesis and immune evasion [6, 32]. Their remarkable plasticity, when hijacked by tumor cells, drives complex TME interactions often linked to poor clinical outcomes, particularly through the activity of M2-like macrophages [33–35]. Nevertheless, M2-like macrophages also contribute to tissue repair, immune resolution, and regulation of excessive inflammation, suggesting that their functions extend beyond immune suppression to maintaining microenvironmental balance. As the dominant infiltrating immune population in the glioma TME, TAMs are key regulators of immune homeostasis, inflammation, and tissue remodeling. They actively shape tumor evolution, spatial architecture, and therapeutic resistance. This extensive heterogeneity and functional diversity of TAMs suggest that broad depletion strategies may be counterproductive, underscoring the need for selective reprogramming approaches to restore antitumor immunity.

The involvement of endogenous FUOM in non-transformed cells within the glioma microenvironment contributes significantly to glioma progression. Reduced FUOM expression in glioma enhances macrophage polarization toward the M2 phenotype, whereas in vivo FUOM depletion exerts potent anti-glioma effects mediated through M2-like macrophages. Meanwhile, our research identifies CXCL13 as a key factor regulated by FUOM in glioma, essential for recruiting and sustaining the M2-like macrophage phenotype, which in turn suppresses glioma progression. M1-like macrophage activation represents the initial inflammatory response, while M2-like macrophage polarization may occur subsequently as part of a compensatory or resolution process. In our primary glioma model, which likely reflects a complex inflammatory microenvironment, where pro-inflammatory (M1) and anti-inflammatory (M2) macrophage populations might both be activated in response to stimulus. However, we observed that down regulation of FUOM treatment markedly reduced tumor cell proliferation and overall tumor burden, which is consistent with the prolonged patient survival. Therefore, we interpret the rise in M2 macrophages as a secondary adaptive response following tumor regression, reflecting a shift toward immune homeostasis rather than enhanced tumor growth. Importantly, CXCL13 chemokines regulate the migration of M2-like macrophages, and an increase in these macrophages correlates with higher malignancy in human gliomas, emphasizing the therapeutic potential of targeting TAMs. A previous study showed that blocking CXCR4 (downstream effector of CXCL13) in mouse and human GBM inhibits GBM proliferation. Additionally, systemic delivery of a CXCR4 inhibitor in combination with radiotherapy directly activates macrophages, leading to an effective anti-GBM adaptive immune response through a less suppressive tumor immune microenvironment [36].

Nevertheless, there still exist limitations in our present study. First, although GBM cell lines effectively model the biology of high-grade gliomas, further research using LGG models and normal astrocytic or neuronal controls is needed to generalize these findings across all glioma subtypes. In addition, the CXCL13 chemotaxis effect estimates derived from the cell co-culture model are based on interventional and prospective observational studies, which may be subject to bias and confounding. These factors could have influenced our model estimates and reduced their clinical relevance, underscoring the need for future in vivo studies to validate the findings and elucidate underlying mechanisms.

Our findings indicate that modifying FUOM expression significantly influences the tumor-promoting activities of TAMs and the malignant progression of glioma. Our present study proposes an innovative therapeutic strategy for glioma that targets both the tumor and immune cells in the brain TME, which goes beyond the conventional method of targeting glioma cell elimination. Our proposed strategy involves altering the function of macrophages, which are usually central to therapies aimed at the tumor microenvironment, to not just nullify the tumor-promoting effects of macrophages but also to actively convert them into agents that can suppress tumor growth. Observations of comparable signaling mechanisms in human GBM support the theory that this intervention could be clinically beneficial, particularly for patients with gliomas that do not express FUOM.

## Supplementary information

Supplemental Figure Legends

Supplemental Figure 1

Supplemental Figure 2

Supplemental Figure 3

Supplemental Figure 4

Supplemental Table 1

Supplemental Table 2

Supplemental File 1

Supplemental File 2

Informed Consent Statement

## Data Availability

The original contributions presented in the study are included in the article. Further inquiries can be directed to the corresponding authors.
