# Supplementary material for: Targeting the tumor microenvironment: reprogramming macrophages as a novel therapeutic strategy in FUOM-deficient glioblastoma
Source: Cell Death Dis. 2026 Apr 9;17(1):500. doi: 10.1038/s41419-026-08701-5 (PMC13187179; doi:10.1038/s41419-026-08701-5)
Supplement: Supplementary file 1 — Supplemental Figure Legends [file 41419_2026_8701_MOESM1_ESM.docx]

**Supplemental Figure 1. FUOM mRNA expression related to glioma patient prognosis.**

A. Image of FUOM protein expression on non-tumor patient brain tissues (Scale bar = 100um).

A1. Magnified region of image A showing distribution and expression of FUOM on non-tumor patient brain tissue (Scale bar = 20um).

B. Image of FUOM protein expression on LGG patient glioma TMAs (Scale bar = 100um).

B1. Magnified region of image B showing distribution and expression of FUOM on LGG patient glioma tissue (Scale bar = 20um).

C. Image of FUOM protein expression on GBM patient glioma TMAs (Scale bar = 100um).

C1. Magnified region of image B showing distribution and expression of FUOM on GBM patient glioma tissue (Scale bar = 20um).

D. An ROC curve is plotted to display the prognostic accuracy of FUOM. The area under the curve was 0.873 (95% CI: 0.856–0.890).

E. FUOM mRNA expression in CCGA dataset (*** *P* < 0.005).

F. Kaplan-Meier survival estimates between high and low FUOM expression in CCGA dataset (HR: 1.91, 95% CI: 1.44-2.53, *P* < 0.001).

G-H. FUOM mRNA expression in TCGA dataset (*** P < 0.005).

I. Kaplan-Meier survival estimates between high and low FUOM expression in TCGA dataset (HR: 2.43, 95% CI: 1.74-3.39, *P* < 0.001).

**Supplemental Figure 2. FUOM recombinant protein restores glioma cell proliferation and down-regulation of FUOM promotes CXCL13 releasing through PI-3K signaling pathway.**

A. CCK-8 assay of FUOM down-regulated T98G glioma cell line after full-length recombinant FUOM protein (0.25 ug/uL) treatment (** *P* < 0.01).

B. CCK-8 assay of FUOM down-regulated U251 glioma cell line after full-length recombinant FUOM protein (0.25 ug/uL) treatment (*** *P* < 0.005).

C. ELISA detection of CXCL13 expression upon pathway inhibitors treatment targeting PI3K/AKT (LY294002), ERK (PD98059), and MAPK (Rac1/Rac2 inhibitor, EHT1864) in FUOM-downregulated glioma cell lines (* *P* < 0.05, *** *P* < 0.005).

D, E. Quantification analysis of the migration and invasion abilities of FUOM down-regulated U251 glioma cell line after CXCL13-oe treatment (** *P* < 0.01, *** *P* < 0.005).

F, G. Quantification analysis of the migration and invasion abilities of FUOM down-regulated T98G glioma cell line after CXCL13-oe treatment (** *P* < 0.01, *** *P* < 0.005).

H. CCK-8 assay of FUOM down-regulated U251 glioma cell line after CXCL13-oe treatment (** *P* < 0.01).

I. CCK-8 assay of FUOM down-regulated T98G glioma cell line after CXCL13-oe treatment.

J-N. QPCR testing for down-stream effector molecules upon pathway inhibitors treatment targeting PI3K/AKT (LY294002) (* *P* < 0.05, ** *P* < 0.01, *** *P* < 0.005, **** *P* < 0.0001).

**Supplemental Figure 3. Mouse BMDMs co-cultured with FUOM down-regulated glioma cells exhibit increased migration and phagocytosis functions upon CXCL13 over-expression.**

A. Schematic diagram illustrating the procedures of acquiring mouse bone marrow-derived macrophages.

B, C. Flow cytometric analysis of successfully differentiated F4/80^+^ CD115^+^ BMDMs upon M-CSF-1 stimulation for 4 days.

D-J. Phagocytosis assays of BMDM, M1/M2-BMDMs co-cultured with FUOM-down-regulated glioma cells.

K-M. Quantification analysis of the migration and invasion abilities of FUOM down-regulated U251 glioma cell line/cell supernatant co-cultured with M2-BMDM (*** *P* < 0.005).

N-P. Quantification analysis of the migration and invasion abilities of FUOM down-regulated T98G glioma cell line/cell supernatant co-cultured with M2-BMDM (* *P* < 0.05, **** *P* < 0.0001).

**Supplemental Figure 4. FUOM-shc and/or CXCL13-oe treatment induce high expressed IL-10 and TGF-β expression and polarize macrophages toward M2 phenotype through STAT-3.**

A, B. QPCR testing of IL-10, and TGF-β expression upon THP-1 co-cultured with FUOM-shc+CXCL13-oe treatment glioma cells (T98G, U251) (* *P* < 0.05, *** *P* < 0.005).

C, D. QPCR testing of IL-10, and TGF-β expression upon BMDMs co-cultured with FUOM-shc+CXCL13-oe treatment glioma cells (T98G, U251) (** *P* < 0.01, *** *P* < 0.005).

E. QPCR testing of macrophage M1 polarization regulator upon BMDMs co-cultured with FUOM-shc+CXCL13-oe treatment glioma cells (T98G, U251) (*** *P* < 0.005).

F-H. QPCR testing of macrophage M2 polarization regulators upon BMDMs co-cultured with FUOM-shc+CXCL13-oe treatment glioma cells (T98G, U251) (* *P* < 0.05, ** *P* < 0.01, *** *P* < 0.005).
