# Supplementary figures and images for: Targeting the tumor microenvironment: reprogramming macrophages as a novel therapeutic strategy in FUOM-deficient glioblastoma
Source: Cell Death Dis. 2026 Apr 9;17(1):500. doi: 10.1038/s41419-026-08701-5 (PMC13187179; doi:10.1038/s41419-026-08701-5)

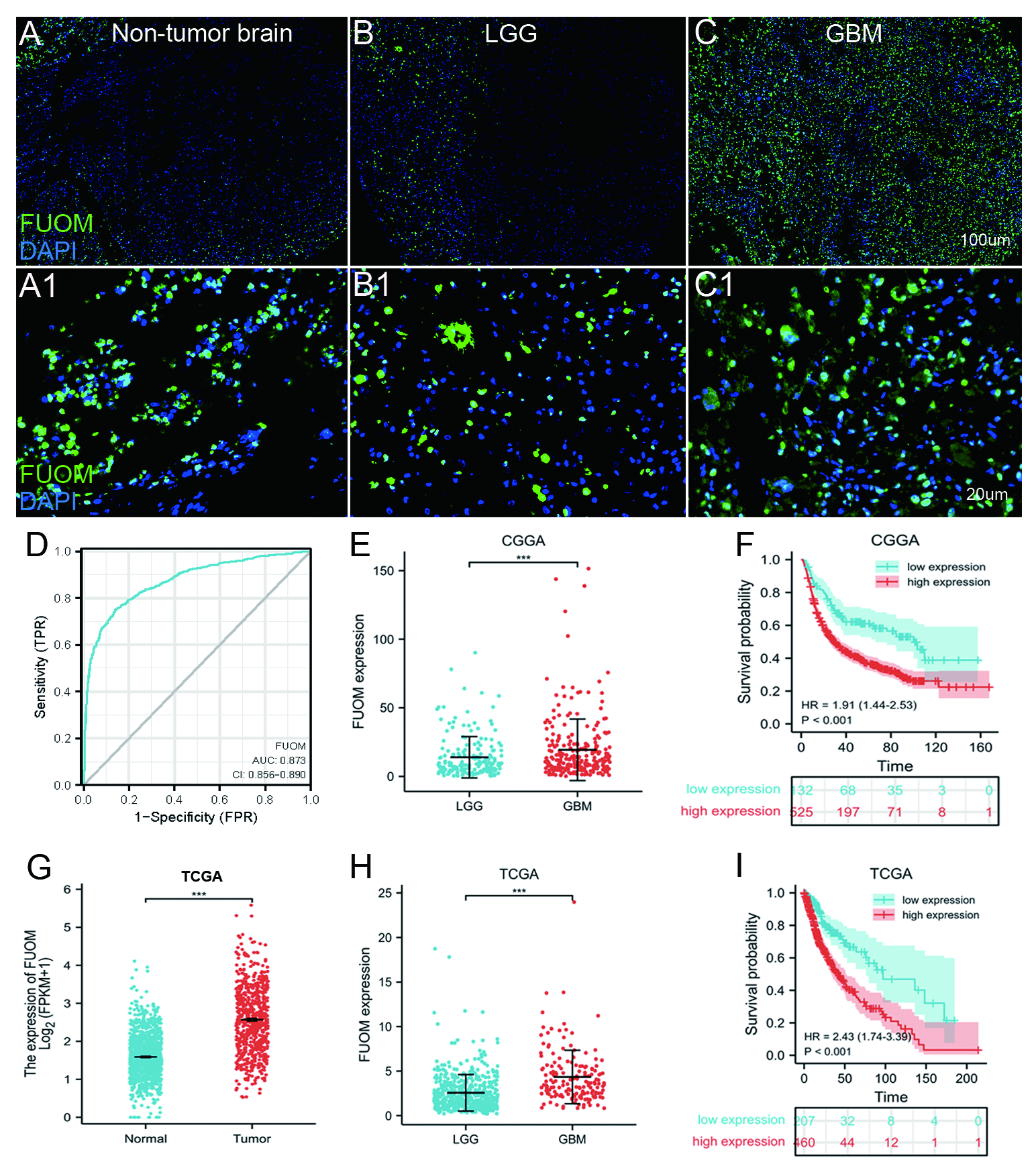

Supplement: Supplementary file 2 — Supplemental Figure 1 [file 41419_2026_8701_MOESM2_ESM.tif]

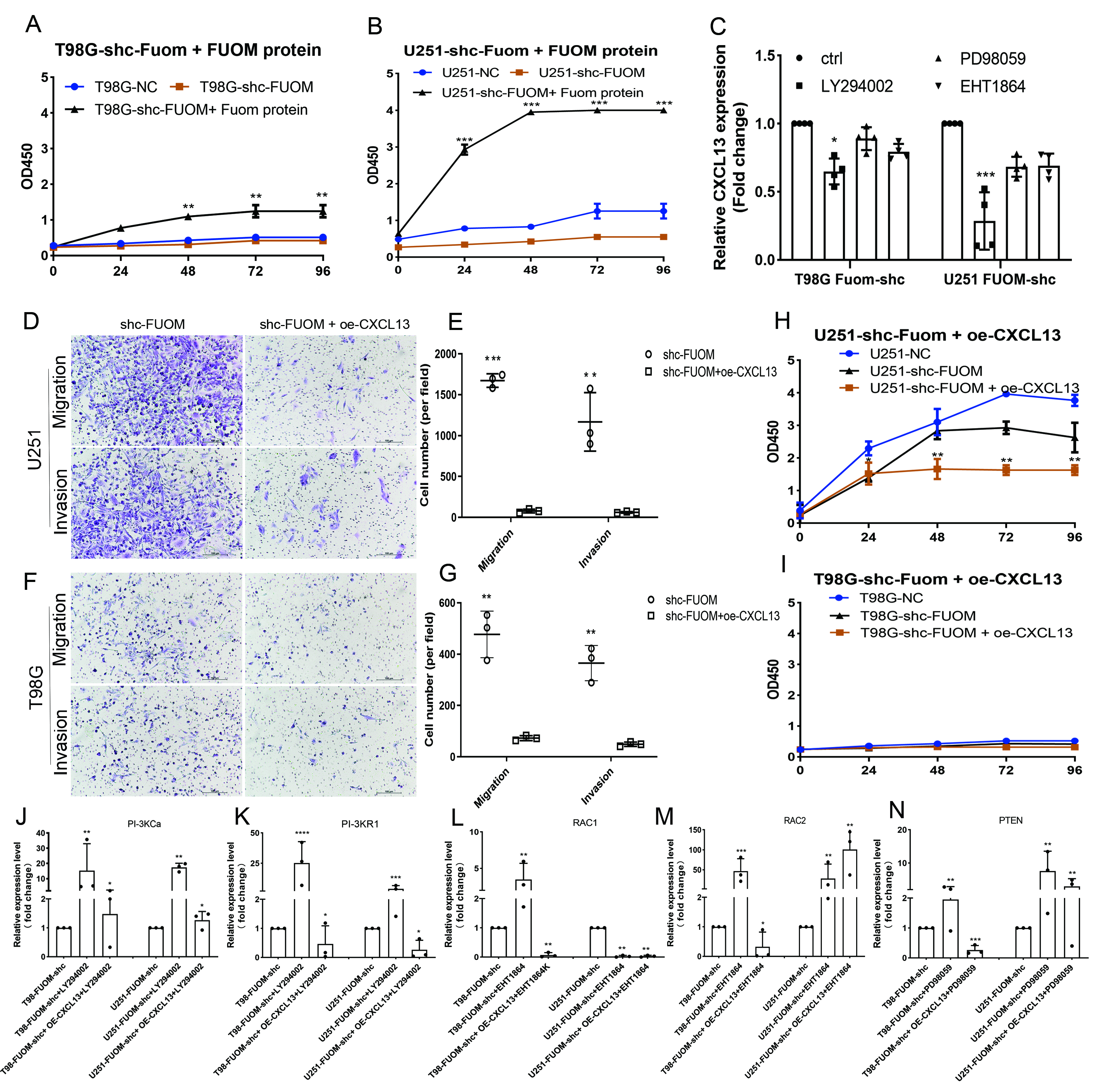

Supplement: Supplementary file 3 — Supplemental Figure 2 [file 41419_2026_8701_MOESM3_ESM.tif]

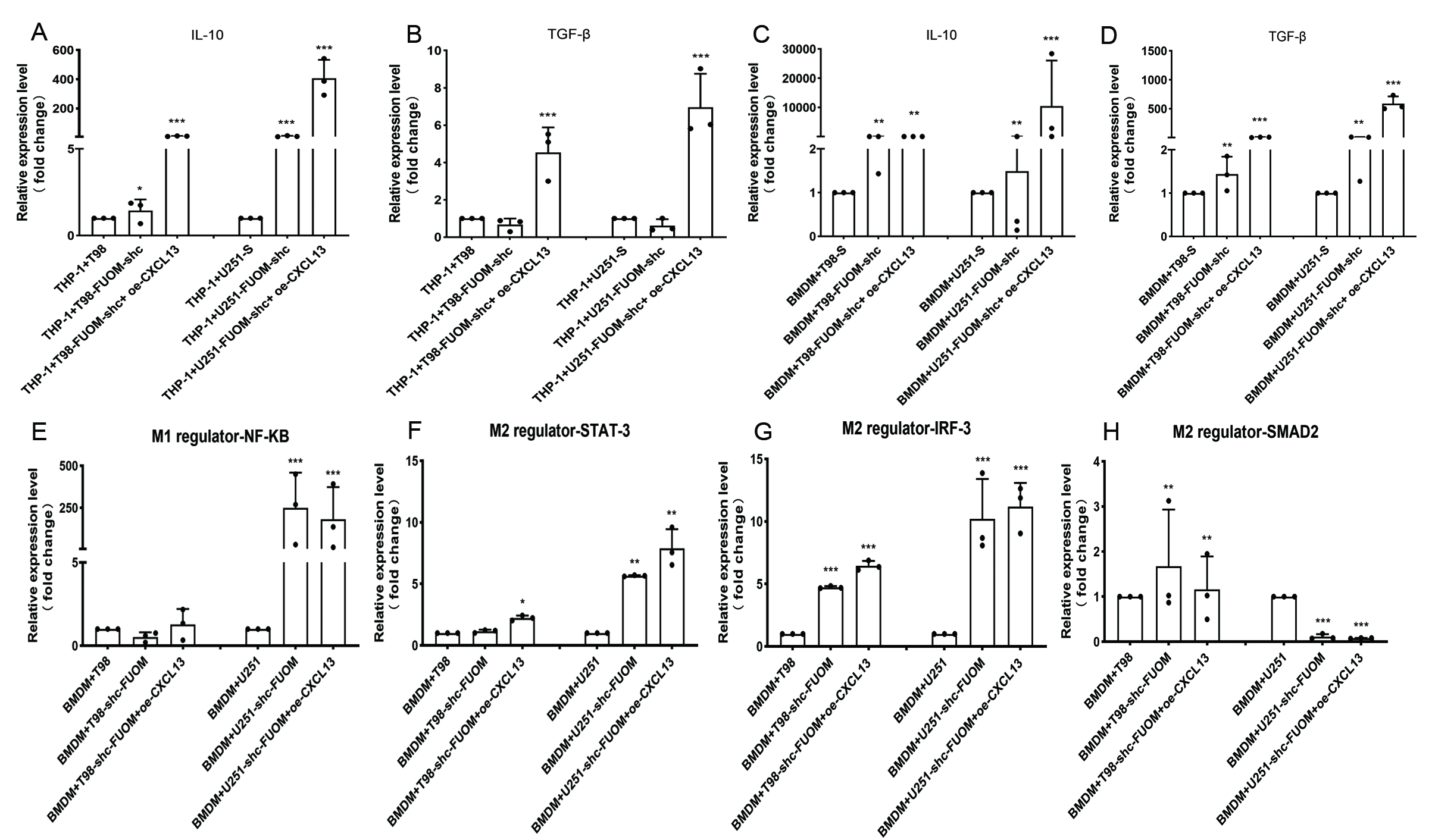

Supplement: Supplementary file 5 — Supplemental Figure 4 [file 41419_2026_8701_MOESM5_ESM.tif]
