# Supplementary material for: Targeting the tumor microenvironment: reprogramming macrophages as a novel therapeutic strategy in FUOM-deficient glioblastoma
Source: Cell Death Dis. 2026 Apr 9;17(1):500. doi: 10.1038/s41419-026-08701-5 (PMC13187179; doi:10.1038/s41419-026-08701-5)
Supplement: Supplementary file 6 — Supplemental Table 1 [file 41419_2026_8701_MOESM6_ESM.docx]

**Supplemental Table. 1. Immune cell markers used in mIHC staining panels**

| **Immune cell type** | **Marker** | **Features** |
| --- | --- | --- |
| **B cell** | CD20 | Mature B cell marker |
| **Treg cell** | CD25 | IL-2 receptor α-chain (high on Tregs) |
| **Neutriphil** | CD66b | Specific for activated neutrophils |
| **DC** | CD83 | Mature DC surface marker (high on mature DCs) |
| **T cell** | CD3 | Pan-T cell marker |
|  | CD4 | Helper T cells |
|  | CD8 | Cytotoxic T cells |
| **Macrophage** | CD68 | Pan-macrophage marker (lysosomal glycoprotein) |
|  | CD86 | M1-type macrophage marker |
|  | CD206 | M2-type macrophage marker |
|  | CD163 | M2-type macrophage marker |
|  | F4/80 | Common macrophage marker in mouse tissues |
