# Supplementary material for: Targeting the tumor microenvironment: reprogramming macrophages as a novel therapeutic strategy in FUOM-deficient glioblastoma
Source: Cell Death Dis. 2026 Apr 9;17(1):500. doi: 10.1038/s41419-026-08701-5 (PMC13187179; doi:10.1038/s41419-026-08701-5)
Supplement: Supplementary file 7 — Supplemental Table 2 [file 41419_2026_8701_MOESM7_ESM.docx]

**Supplemental Table 2. Primer sequences**

| Target gene | Primer sequence （5'-3'） | | Size（bp） |
| --- | --- | --- | --- |
| FUOM | Forward | CTGGACACCTATGTGGAGAGTC | 22 |
|  | Reverse | CTGCGTAGGATGGACTCGTACT | 22 |
| CD163 | Forward | CCAGAAGGAACTTGTAGCCACAG | 23 |
|  | Reverse | CAGGCACCAAGCGTTTTGAGCT | 22 |
| iNOS | Forward | GACTTTCCAAGACACACTTCAC | 22 |
|  | Reverse | GACTTTCCAAGACACACTTCAC | 22 |
| IL10  TGF-β  MMP9 | Forward  Reverse | GTCTCCGAGATGCCTTCACAGA  TCAGACAAGGCTTGGCAACCCA | 22  22 |
|  | Forward  Reverse  Forward  Reverse | TACCTGAACCCGTGTTGCTCTC  GTTGCTGAGGTATCGCCAGGAA  GCCACTACTGTGCCTTTGAGTC  CCCTCAGAGAATCGCCAGTACT | 22  22  22  22 |
| IL-6  IL-1β  TNF-α | Forward  Reverse | AGACAGCCACTCACCTCTTCAG  TTCTGCCAGTGCCTCTTTGCTG | 22  22 |
|  | Forward  Reverse  Forward  Reverse | GCCAGTGAAATGATGGCTTATT  AGGAGCACTTCATCTGTTTAGG  CTCTTCTGCCTGCTGCACTTTG  ATGGGCTACAGGCTTGTCACTC | 22  22  22  22 |
| PIK3CA | Forward  Reverse | CCACGACCATCTTCGGGTG  ACGGAGGCATTCTAAAGTCACTA | 19  23 |
| PIK3R1 | Forward  Reverse | ACACCACGGTTTGGACTATGG  GGCTACAGTAGTGGGCTTGG | 21  20 |
| RAC1 | Forward  Reverse | \| GAGACGGAGCTGTTGGTAAAA \| \| --- \| \| ATAGGCCCAGATTCACTGGTT \| | 22  21 |
| RAC2 | Forward  Reverse | \| GACAGTAAGCCGGTGAACCTG \| \| --- \| \| CTGACTAGCGAGAAGCAGATG \| | 22  22 |
| PTEN | Forward  Reverse | \| TGGATTCGACTTAGACTTGACCT \| \| --- \| \| GCGGTGTCATAATGTCTCTCAG \| | 23  22 |
