# Supplementary material for: Targeting the tumor microenvironment: reprogramming macrophages as a novel therapeutic strategy in FUOM-deficient glioblastoma
Source: Cell Death Dis. 2026 Apr 9;17(1):500. doi: 10.1038/s41419-026-08701-5 (PMC13187179; doi:10.1038/s41419-026-08701-5)
Supplement: Supplementary file 9 — Supplemental File 2 [file 41419_2026_8701_MOESM9_ESM.pptx]

## Slide 1
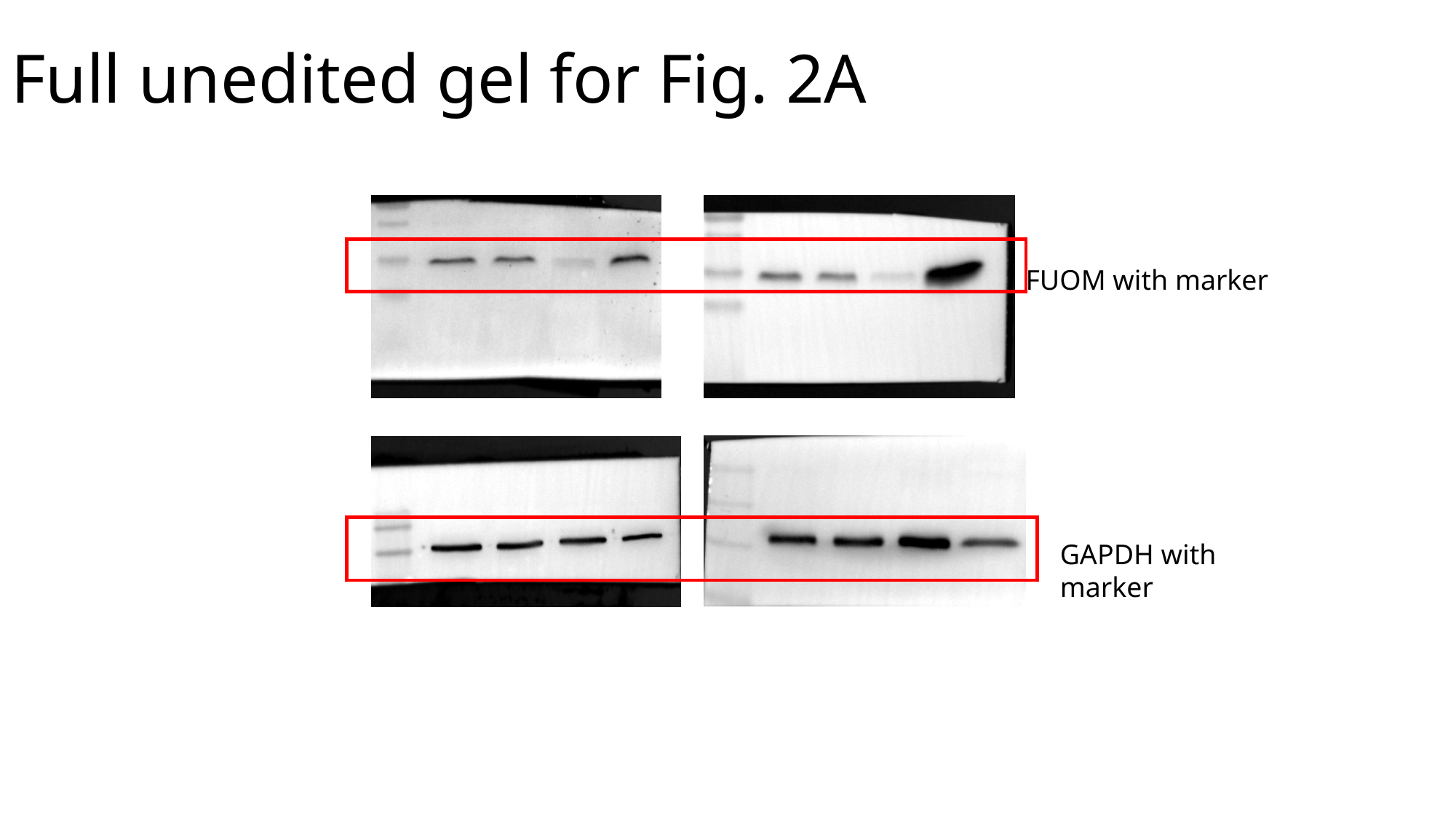

# Full unedited gel for Fig. 2A
FUOM with marker
GAPDH with marker

## Slide 2
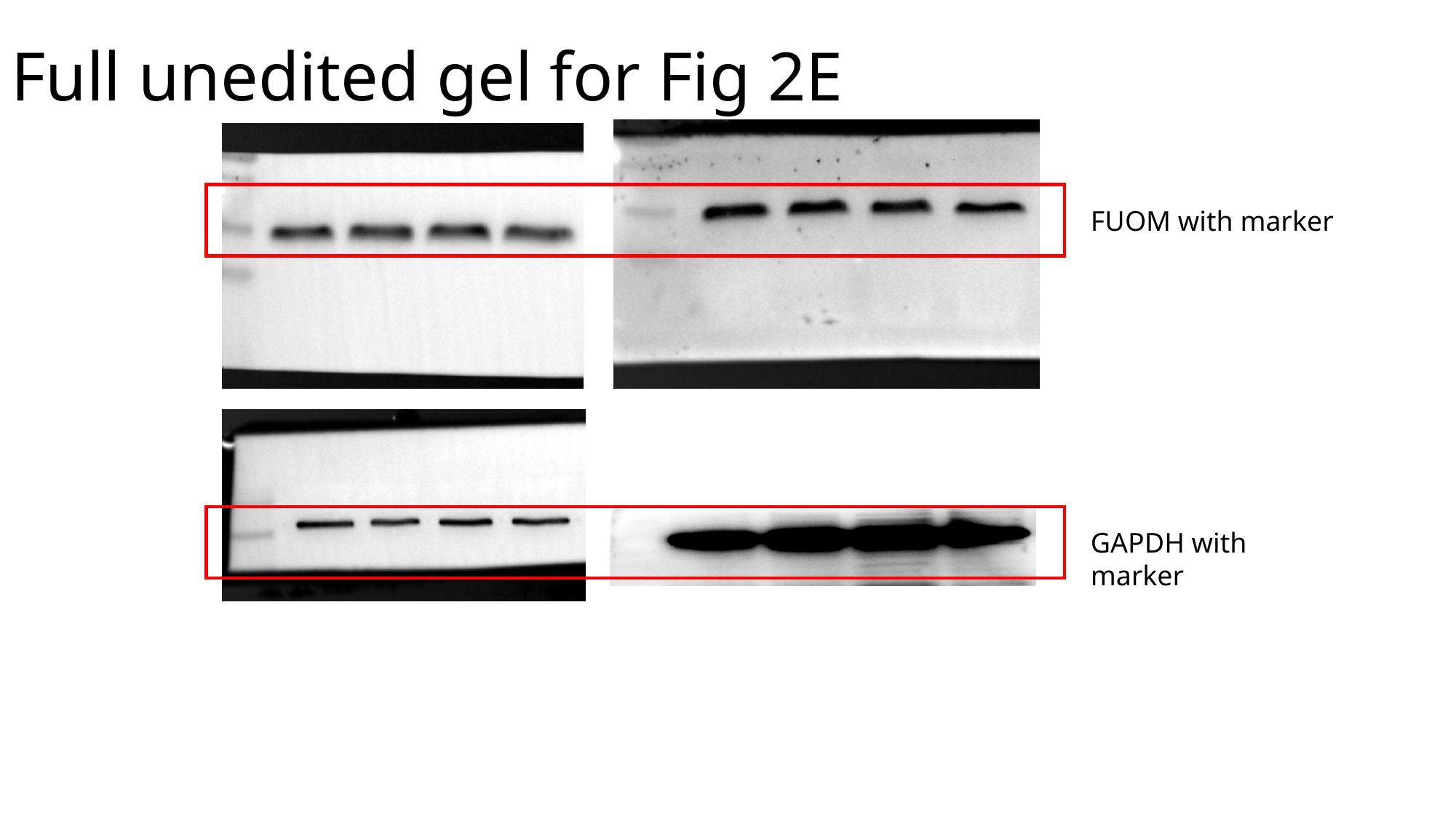

# Full unedited gel for Fig 2E
FUOM with marker
GAPDH with marker

## Slide 3
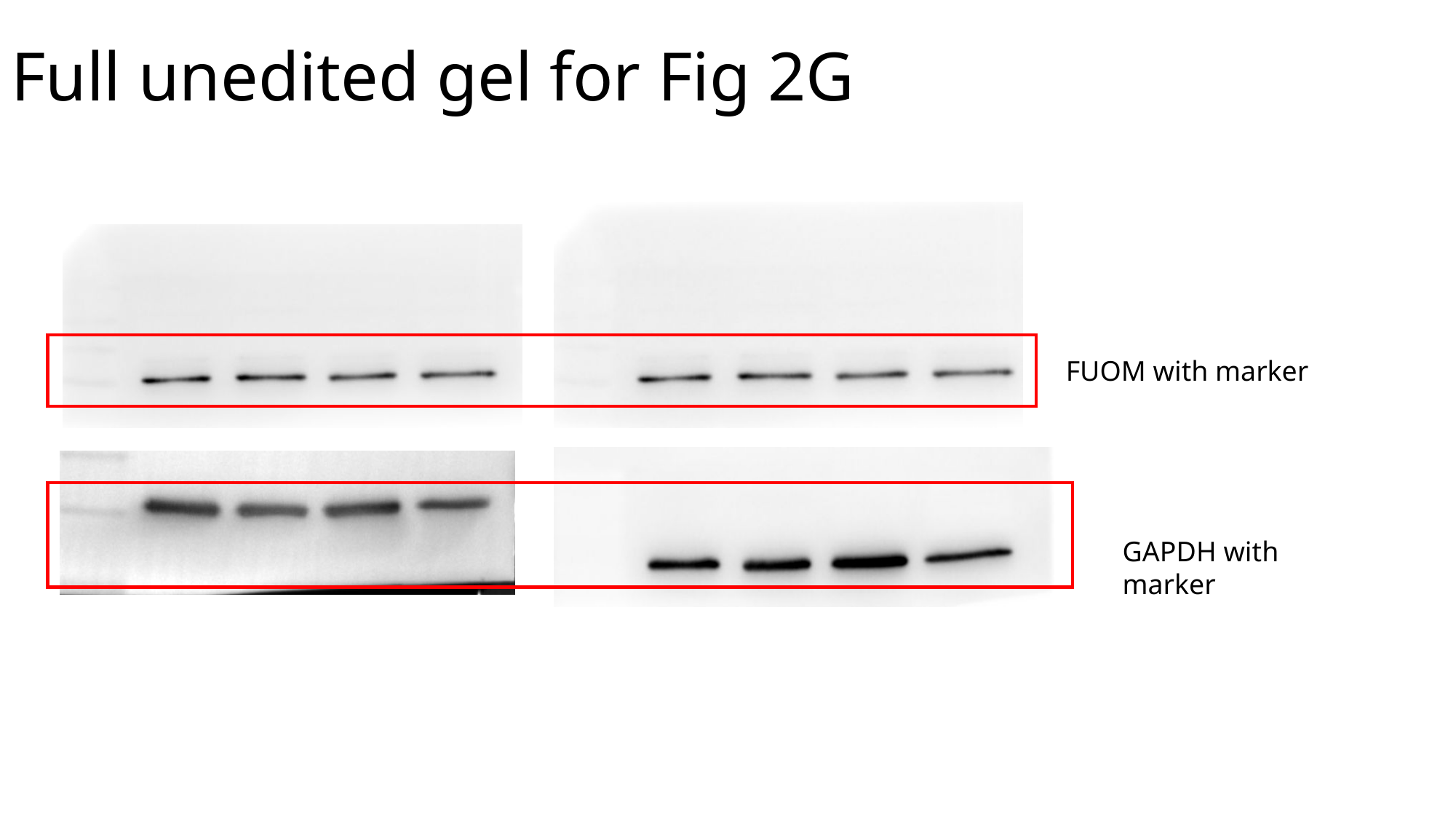

# Full unedited gel for Fig 2G
FUOM with marker
GAPDH with marker

## Slide 4
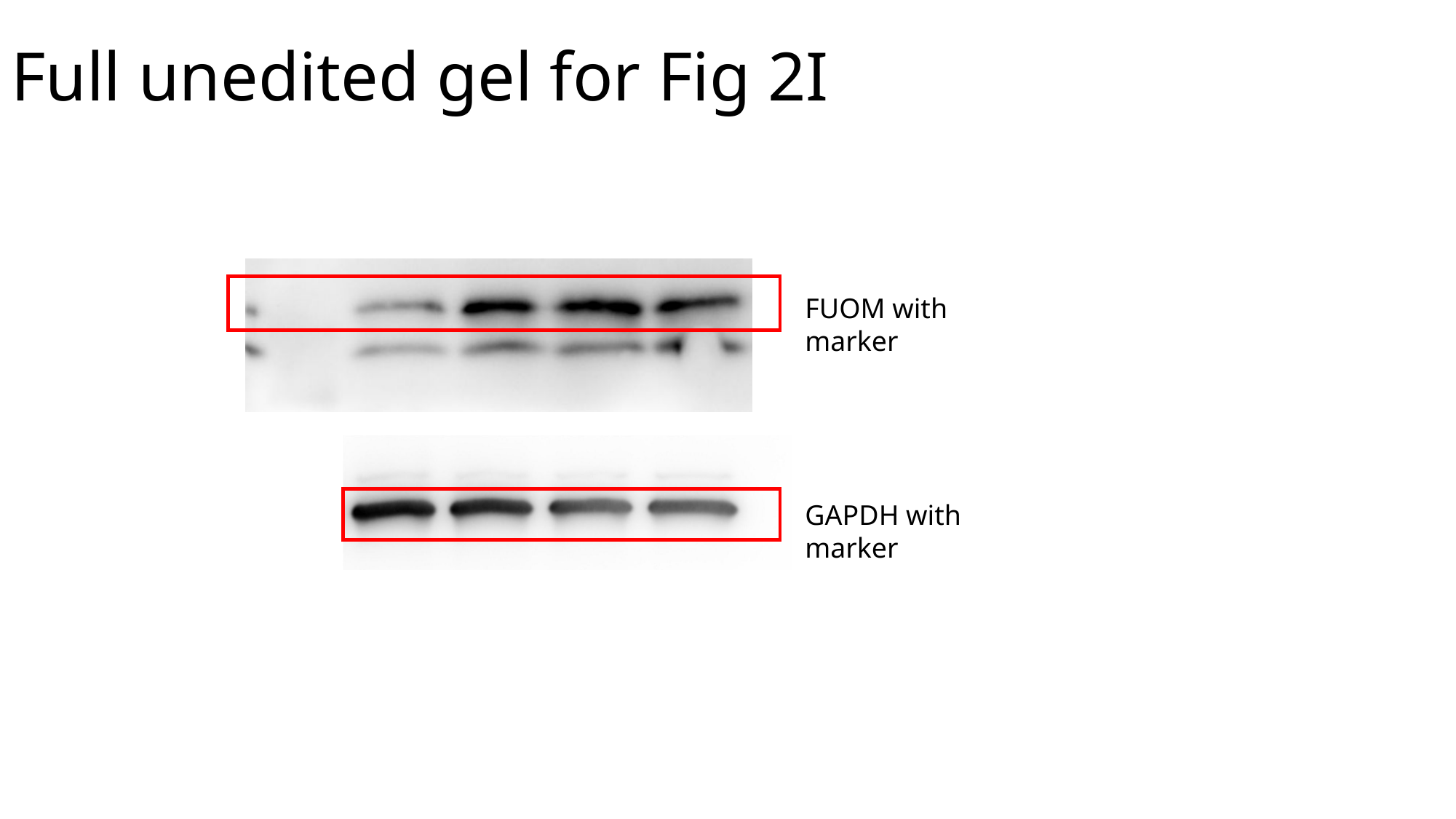

# Full unedited gel for Fig 2I
FUOM with marker
GAPDH with marker

## Slide 5
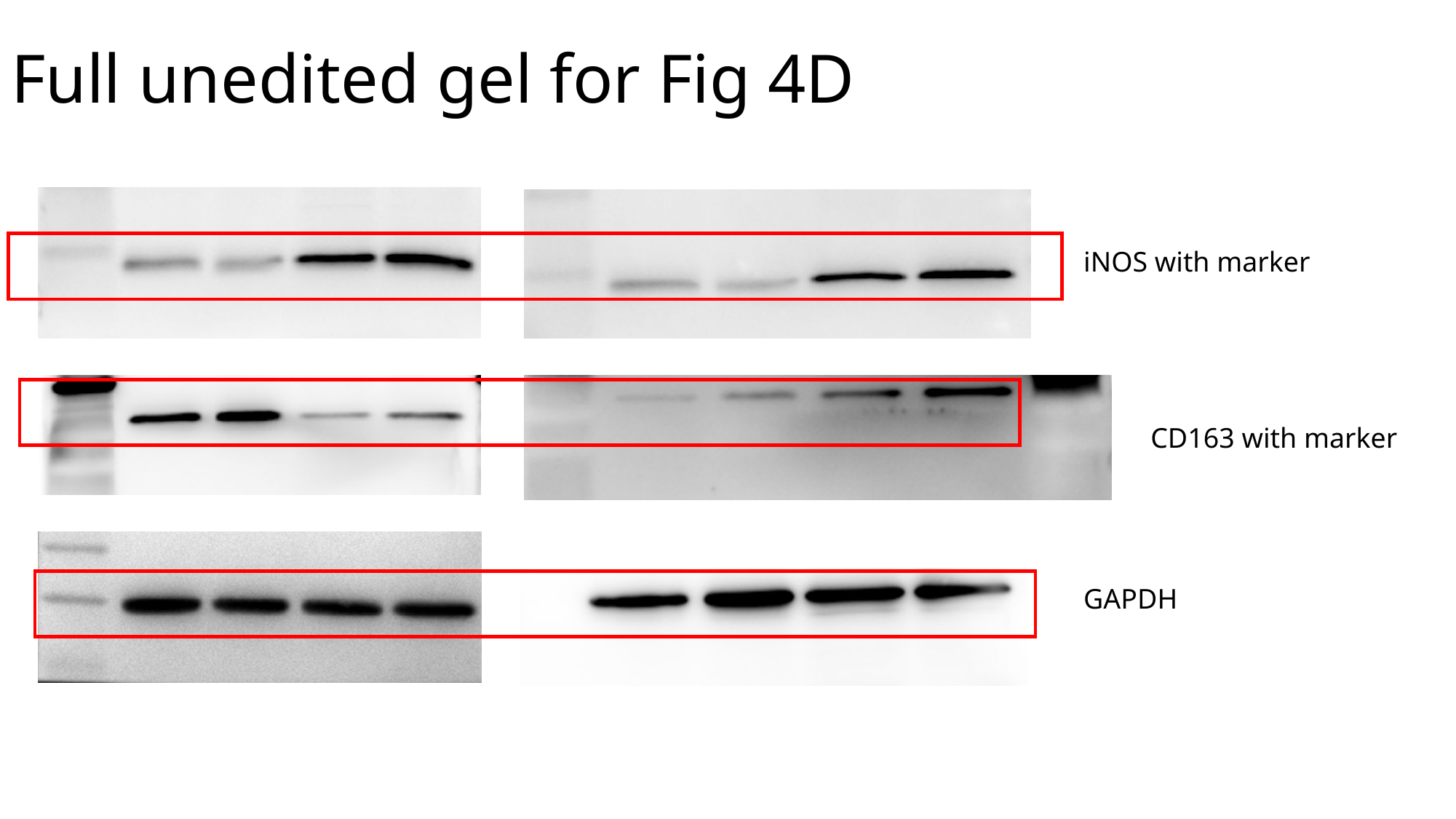

# Full unedited gel for Fig 4D
iNOS with marker
CD163 with marker
GAPDH

## Slide 6
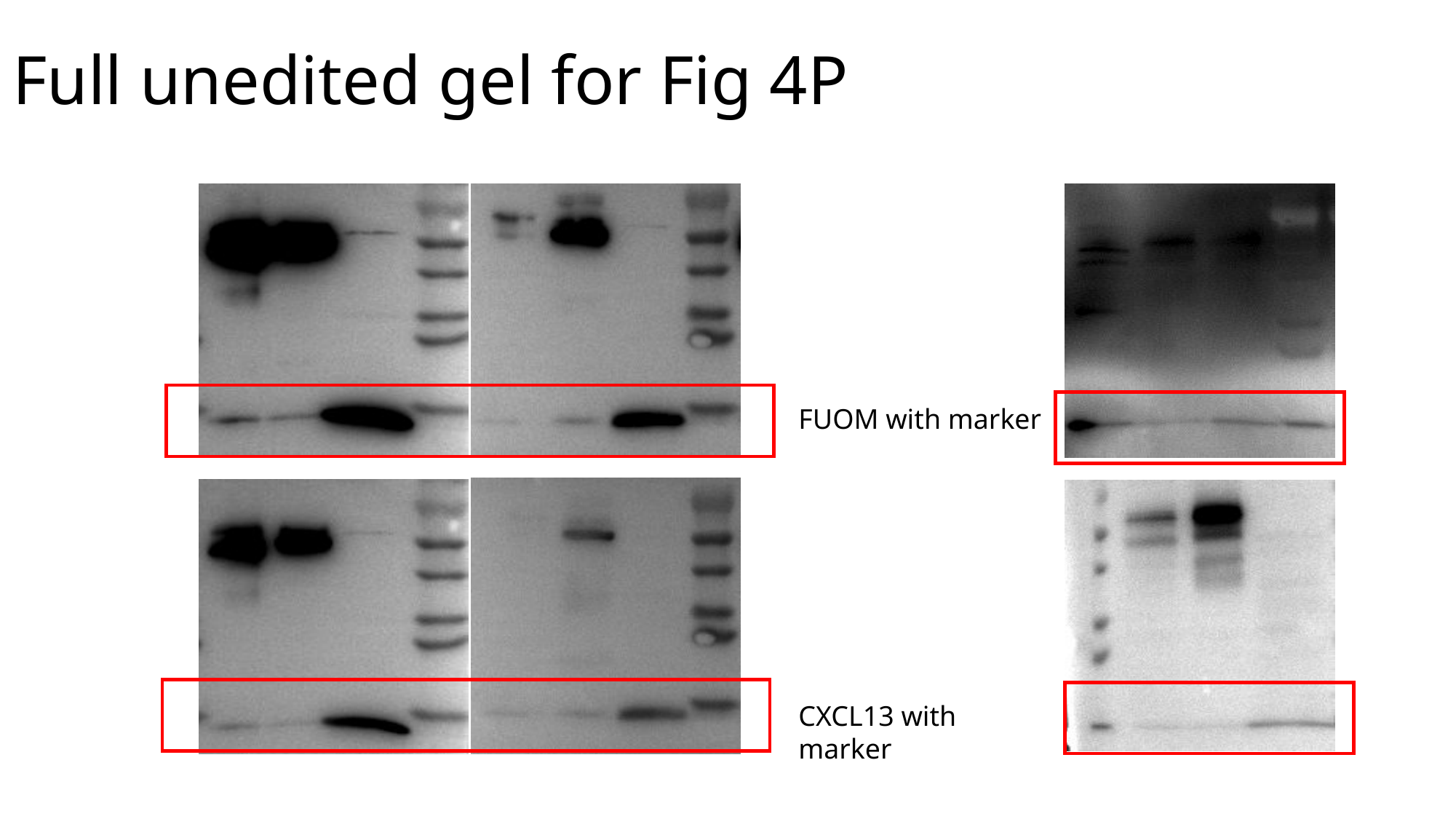

# Full unedited gel for Fig 4P
FUOM with marker
CXCL13 with marker

## Slide 7
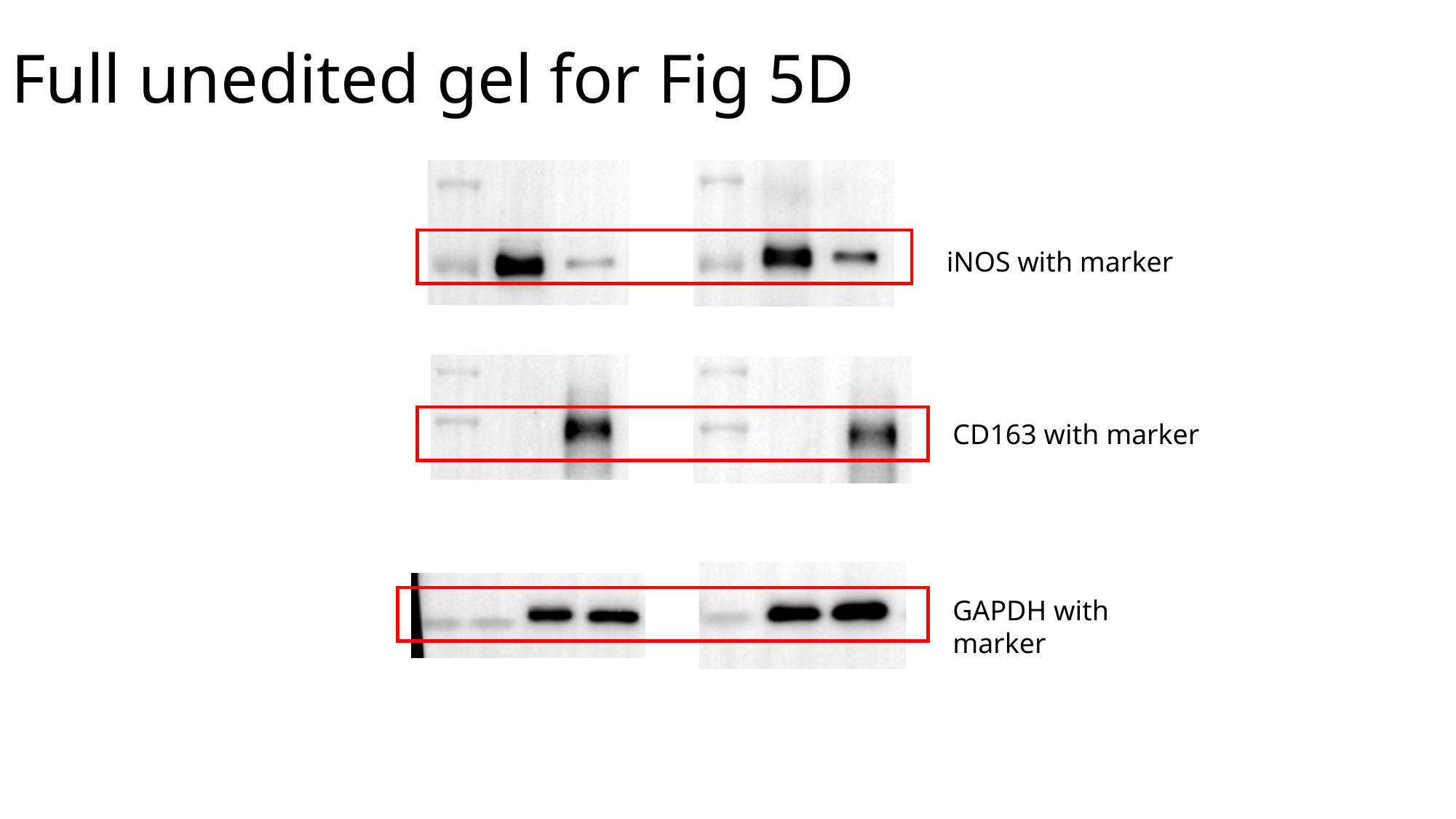

# Full unedited gel for Fig 5D
iNOS with marker
CD163 with marker
GAPDH with marker

## Slide 8
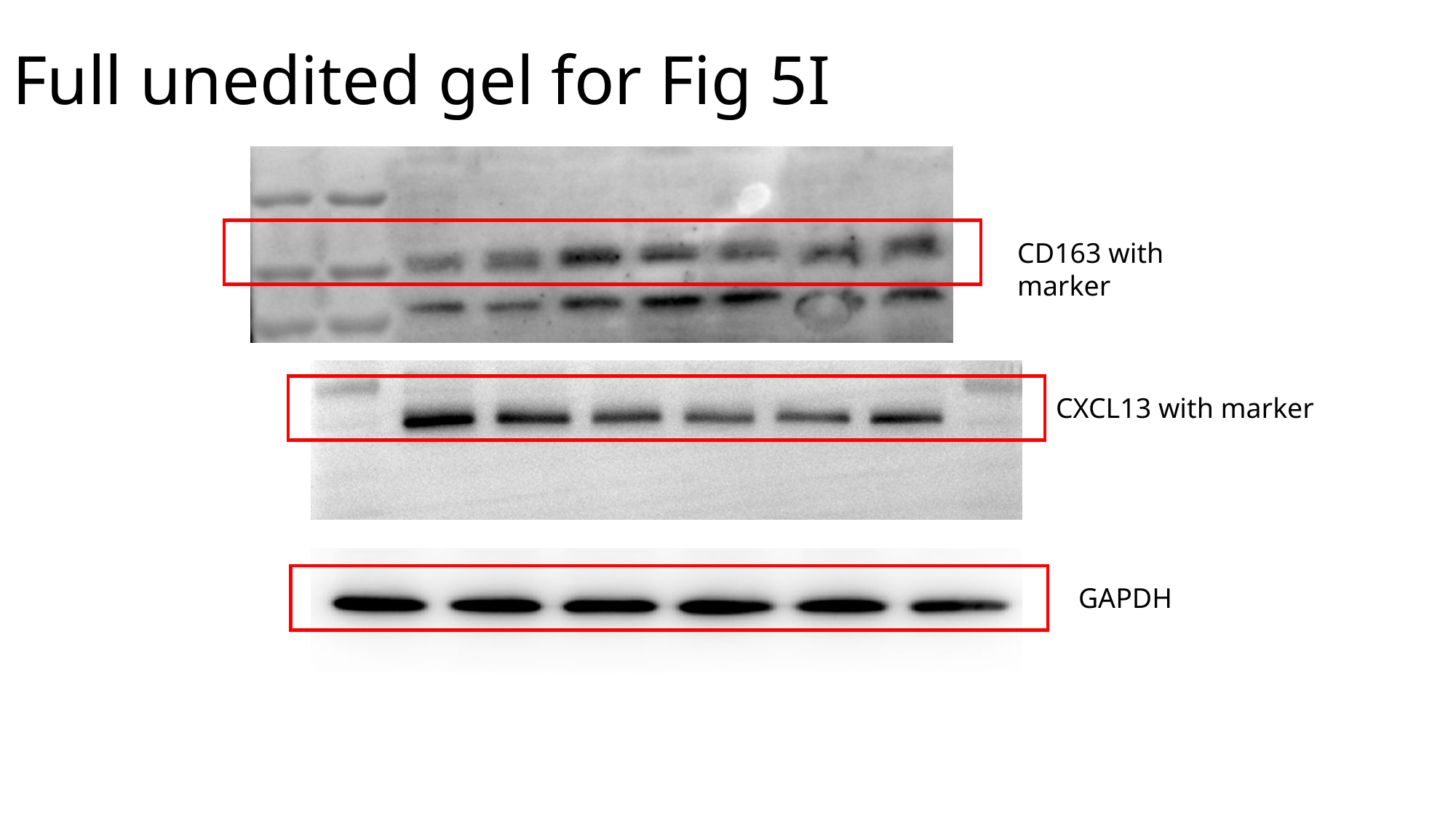

# Full unedited gel for Fig 5I
CD163 with marker
CXCL13 with marker
GAPDH
