# Supplementary material for: Targeting the tumor microenvironment: reprogramming macrophages as a novel therapeutic strategy in FUOM-deficient glioblastoma
Source: Cell Death Dis. 2026 Apr 9;17(1):500. doi: 10.1038/s41419-026-08701-5 (PMC13187179; doi:10.1038/s41419-026-08701-5)
Supplement: Supplementary file 10 — Informed Consent Statement [file 41419_2026_8701_MOESM10_ESM.pdf]

Informed Consent Statement

All clinical samples used in this study were obtained from the Clinical Biobank of the Affiliated Hospital of Nantong University. All samples included in this research were voluntarily donated by patients. Prior to donation, each donor received comprehensive information about the purpose, procedures, potential benefits, and risks associated with the collection and use of their specimens, and written informed consent forms are securely maintained in the records of the Clinical Biobank of the Affiliated Hospital of Nantong University. Use of the samples involving human participants were approved by the Bioethics Committee of the Affiliated Hospital of Nantong University (No. 2018-K020) and with the 1964 Helsinki Declaration and its later amendments or comparable ethical standards.

The corresponding Biobank contact and approval reference number is provided below.

Authorized representative Name: Jianfei Huang

Signature: [illegible] Date: 2026.3.18

伦理审查批件

审查编号：2018-K020

审查日期	2018 年 03 月 13 日		
审查会议地点	药物临床试验机构会议室		
研究项目名称	恶性实体肿瘤诊疗新靶点的筛查及部分作用机制研究		
审查文件	1. 伦理审查申请表；<br>2. 研究方案（V1.0/2018.02.01）；<br>3. 免除知情同意申请；<br>4. 研究者履历；		
研究科室	生物样本库	主要研究者	黄剑飞
申办者/项目来源	自选课题		
伦理审查方式	√会议审查　□ 快速审查	审查类别	初始审查
投票结果	应到人数：15 人　实到人数：10 人　弃权人数：0 人<br>回避委员：0 人　**投票结果：** 同意 10 票		

审查意见

根据卫计委《涉及人的生物医学研究伦理审查办法》（2016）、SFDA《药物临床试验质量管理规范》（2003）、CFDA《医疗器械临床试验质量管理规范》（2016）、WMA《赫尔辛基宣言》（2013）和 CIOMS《人体生物医学研究国际道德指南》等相关文件的伦理原则，经本伦理委员会审查，同意按所批准的临床研究方案、免除知情同意开展本研究。

请遵循 GCP 原则、遵循伦理委员员批准的方案开展临床研究，保护受试者的健康与权利。

研究过程中若变更主要研究者，对临床研究方案、知情同意书、招募材料等的任何修改，请申请人提交修正案审查申请。

发生严重不良事件，请申请人及时提交严重不良事件报告。

请按照伦理委员会规定的年度/定期跟踪审查频率，申请人在截止日期前 1 个月提交研究进展报告。

研究纳入了不符合纳入标准或符合排除标准的受试者，符合中止试验规定而未让受试者退出研究，给予错误治疗或剂量，给予方案禁止的合并用药等没有遵从方案开展研究的情况；或可能对受试者的权益/健康以及研究的科学性造成不良影响等违背 GCP 原则的情况，请申办方/监查员/研究者提交违背方案报告。

申请人暂停或提前终止临床研究，请及时提交暂停/终止研究报告。

完成临床研究，请申请人提交研究完成报告。

年度/定期跟踪审查频率	□6 个月　√1 年　□不适用
批件有效期	十年（2018.03.14-2028.03.13）
主任委员签字	[signature]
伦理委员会	南通大学附属医院伦理委员会
日期	2018 年 03 月 14 日

伦理委员会联系电话：0513-85052390；E-mail：lunlib@126.com
